# HIV-1 Capsid Uncoating Is a Multistep Process That Proceeds through Defect Formation Followed by Disassembly of the Capsid Lattice

**DOI:** 10.1021/acsnano.3c07678

**Published:** 2024-01-19

**Authors:** Levi B. Gifford, Gregory B. Melikyan

**Affiliations:** † Department of Pediatrics, Emory University School of Medicine, Atlanta, Georgia 30322, United States; ‡ Children’s Healthcare of Atlanta, Atlanta, Georgia 30322, United States

**Keywords:** HIV-1 capsid, single virus tracking, genetic code expansion, click labeling, dynamics of capsid uncoating, nuclear import

## Abstract

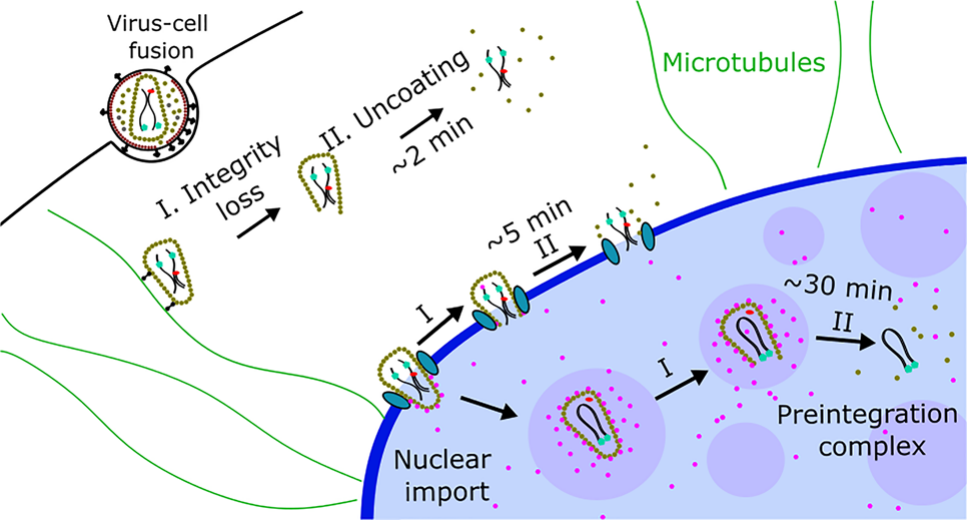

The HIV-1 core consists of a cone-shaped capsid shell made of capsid protein (CA) hexamers and pentamers encapsulating the viral genome. HIV-1 capsid disassembly, referred to as uncoating, is important for productive infection; however, the location, timing, and regulation of uncoating remain controversial. Here, we employ amber codon suppression to directly label CA. In addition, a fluid phase fluorescent probe is incorporated into the viral core to detect small defects in the capsid lattice. This double-labeling strategy enables the visualization of uncoating of single cores *in vitro* and in living cells, which we found to always proceed through at least two distinct steps—the formation of a defect in the capsid lattice that initiates gradual loss of CA below a detectable level. Importantly, intact cores containing the fluid phase and CA fluorescent markers enter and uncoat in the nucleus, as evidenced by a sequential loss of both markers, prior to establishing productive infection. This two-step uncoating process is observed in different cells, including a macrophage line. Notably, the lag between the release of fluid phase marker and terminal loss of CA appears to be independent of the cell type or reverse transcription and is much longer (>5-fold) for nuclear capsids compared to cell-free cores or cores in the cytosol, suggesting that the capsid lattice is stabilized by capsid-binding nuclear factors. Our results imply that intact HIV-1 cores enter the cell nucleus and that uncoating is initiated through a localized defect in the capsid lattice prior to a global loss of CA.

The HIV-1 capsid is a large (∼60 nm wide end, ∼40 nm narrow end, ∼100 nm length) proteinaceous structure that is comprised of ∼250 capsid protein (CA) hexamers and exactly 12 pentamers to form the conical capsid lattice.^1−4^ Fusion of HIV-1 with the cell membrane releases the capsid into the cytosol where it interacts with a multitude of cellular dependency and restriction factors. Interactions with host dependency factors promote microtubule transport, import through the nuclear pore complex (NPC), and translocation to nuclear speckles for integration within the speckle-associated genomic domains.^3,5−9^ Capsid disassembly, referred to as uncoating, is required for the release of the HIV-1 preintegration complex, but the extent and cellular sites of CA loss remain controversial.^1,3,10^ HIV-1 capsid stability is tightly regulated by multiple host factors, such as IP6, Sec24C, Nup153, and several molecular motors.^11−14^ Optimal core stability is essential for nuclear import and delivery of viral complexes to the sites of integration, as evidenced by the compromised infectivity of HIV-1 containing CA mutations that hyperstabilize or destabilize the capsid lattice.^2,15−18^ Thus, timely HIV-1 uncoating is critical for productive infection.

HIV-1 uncoating has been traditionally studied using biochemical and functional assays that yielded discrepant findings regarding the sites and timing of capsid disassembly.^19−23^ More recently, single virus imaging in living cells has been employed to visualize the sites and timing of uncoating in the context of productive infection.^6,24−27^ However, contradicting results regarding the sites of productive uncoating have been reported using single virus imaging approaches in live and fixed cells.^6,24−34^ Models proposed based upon imaging, biochemical and functional experiments place HIV-1 uncoating in all three possible cellular compartments: the cytosol, shortly after viral fusion,^19−21,24,35^ the nuclear pore complex,^28−30,32,36^ and the nucleoplasm,^25,27,33,34,36−39^ with terminal disassembly near the sites of integration.^25,27^

Recent correlative light and electron microscopy (CLEM) and electron tomography (ET) experiments have detected cracked capsid lattices and, in rare cases, intact-looking capsids within the nucleus of cells.^33,38^ Although a link between seemingly intact capsid cores in the nucleus and infection could not be established, this finding implies that cores may not lose the capsid lattice upon translocation through the NPC. These CLEM results are consistent with the recent finding that the diameter of intact nuclear pore is not ∼40 nm, as was previously thought, but 60–64 nm, which can, in principle, allow for passage of the entire capsid into the nucleus.^38,40^ These results are also in line with biochemical and genetic evidence suggesting that, at least a fraction of capsid lattice, is preserved in the nucleus^41^ and that viral cores undergo remodeling upon nuclear import.^42−44^ The presence of cracked cores within the nucleoplasm lacking electron density (which likely corresponds to cores without vRNP) has been taken to indicate that uncoating does not culminate in full disassembly of capsid lattice.^33,38^

These controversial findings are likely due to the paucity of minimally invasive CA-labeling approaches that do not majorly affect the virus functionality. Because the HIV-1 capsid is a large and highly complex structure that interacts with multiple host dependency factors and plays critical roles in early infection,^2,3,39,45^ most mutations disrupt the capsid morphology, stability and/or reduce infectivity (e.g.,^2,46^). For this reason, several studies have relied on indirect capsid markers to investigate capsid uncoating. One indirect labeling approach utilizes capsid entrapped GFP content marker derived from the HIV-1 Gag construct with “internal” GFP (iGFP).^24,27,47,48^ Release of a small portion of iGFP trapped in intact HIV-1 cores has been used as a proxy for uncoating.^24,27,48^ However, single particle imaging experiments using this core content marker by two groups led to contradicting conclusions regarding the sites of HIV-1 uncoating.^24,27^ Another indirect HIV-1 capsid marker, cyclophilin A-DsRed (CDR), utilized cyclophilin A that is rendered tetrameric through fusion to DsRed protein and that tightly binds to the cyclophilin A binding loops of capsid through multiple cyclophilin A moieties.^6,26,30,49^ We have previously observed loss of this marker after virus docking at the NPC, prior to nuclear import and productive infection.^6,26,30,49^

Direct capsid labeling approaches include a tetracysteine-tagged^32,50^ or eGFP-tagged CA.^25,32,44^ However, both approaches compromise the infectivity and require a large (10–15-fold) excess of WT CA to produce functional virions. Additionally, nonspecific labeling and significant photobleaching of the tetracysteine tag during live-cell imaging limit its utility.^32,50^ By tracking HIV-1 cores containing the eGFP-CA probe, Burdick and coauthors have observed nuclear import of HIV-1 core without detectable loss of eGFP-CA, followed by loss of the capsid marker in the nucleus, near the site of integration.^25^ More recently, amber codon suppression^51−55^ has been utilized to label the capsid with noncanonical amino acids conjugated to organic fluorophores.^34^ Insertion of a noncanonical amino acid at the alanine-14 position of CA produced infectious viruses, but delayed nuclear import of HIV-1 complexes by roughly 6 h. Comparison of labeled capsid signals in the nuclei of fixed cells and to cell-free labeled cores on coverslips revealed no differences, further supporting the notion that intact cores can enter the nucleus.^34^

To investigate the site(s) and dynamics of capsid uncoating, we sought to directly label the HIV-1 capsid with small organic fluorophores. Toward this goal, we utilized amber codon suppression to label CA at the isoleucine 91 site within the cyclophilin A binding loop. When mixed with comparable amounts of wild-type (WT) CA, the I91 CA mutant containing virions exhibit infectivity close to that of unlabeled pseudoviruses, while allowing for direct labeling of viral capsid and tracking single core uncoating events that result in productive infection. Along with amber codon suppression-based labeling, we produced dual-labeled HIV-1 pseudoviruses containing the fluid phase viral content marker Gag-iYFP (similar to Gag-iGFP^24,27,47,48^) to visualize the relationship between the initial loss of lattice integrity and terminal disassembly of single capsid cores in live cells. With this system, we show that, both the initial loss of integrity and terminal disassembly of capsid occur in the nucleus, in agreement with the recent model for nuclear uncoating.^25,27^ We also find that terminal capsid disassembly is temporally linked to loss of lattice integrity, where the formation of initial/small defect(s) eventually culminates in capsid uncoating. The two-step uncoating phenotype is observed in both osteosarcoma cells and THP-1 macrophage line. We also find that two-step uncoating occurs in the cytosol and after docking at the nuclear pore complex, albeit with shorter lag time between loss of integrity and capsid disassembly. Our results provide important insights into the dynamics of capsid uncoating that progresses through at least one distinct intermediate step preceding terminal loss of CA. These findings are essential for understanding the mechanisms of regulation of the HIV-1’s capsid stability which is critical for productive infection.

## Results

### Selection of Amber Suppression Sites on the Capsid

To directly label the HIV-1 capsid, we genetically tagged the CA protein at various sites using amber codon suppression.^51−56^ We incorporated the noncanonical amino acid (NCAA) trans cyclooctene lysine (TCO) at the A14 residue within the N-terminus of CA, since a previous study reported successful incorporation of NCAAs at this positions without a major effect on infectivity.^34^ Additional sites at positions P85, I91, and G94 in the flexible cyclophilin A binding loop of CA were chosen based on their solvent accessibility (Figure S1). The I91 site is particularly attractive because it is not essential for CypA binding.^57^ Upon pseudovirus production using the mutant/WT CA plasmid ratio of 1.5:1 and labeling with tetrazine-dyes, the I91 site had the least effect on specific infectivity compared to other mutants (Figure S2A). This plasmid ratio was selected to limit the reduction in the I91 mutant infectivity to <2-fold, compared to CA^WT^ (Figure S2B). Using the 1.5:1 mutant:WT ratio, we found that the I91 mutant allowed the best prGag cleavage efficiency compared to the A14, P85, and G94 mutants (Figure S2C), with Gag precursor cleavage being proportional to the fraction of WT CA incorporated into virions (Figure S2D). Having shown that I91 mutation exhibits the least disruption to specific infectivity and prGag cleavage efficiency, we chose I91 for all further single HIV-1 uncoating *in vitro* and in live cells.

### Characterization and Validation of Direct HIV-1 Capsid Protein Labeling with Unnatural Amino Acids

**Figure 1:**
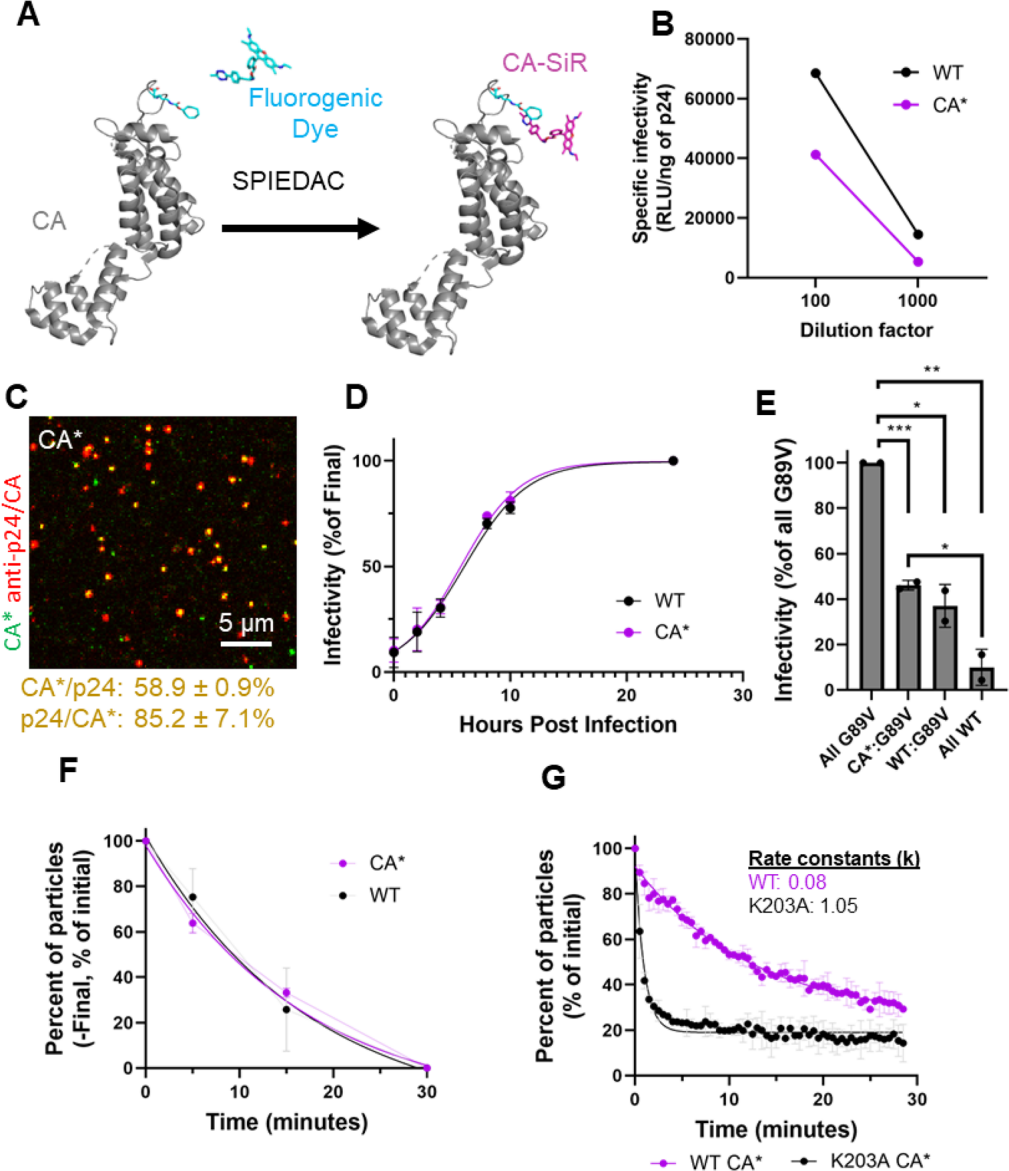
Validation of HIV-1 CA*-labeled pseudoviruses. (A) Schematic illustration of CA protein with the isoleucine-91 site labeled with SiR-tetrazine. PDB models used are 3MGE, 5KAX, 6AAO, and 6NJ4. **(B)** Representative plots for specific infectivity of control (WT) and SiR-tetrazine labeled CA* pseudoviruses in TZM-bl cells measured as firefly luciferase signal (RLU) and normalized to p24 content. **(C)** Cell-free CA* pseudovirus plated on poly-l-lysine coated coverglass, labeled with 250 nM SiR-tetrazine and immunostained with mouse anti-p24 AG3.0 antibody and antimouse AF568 second antibody. Colocalization is calculated between three independent preps. Mean and SD are listed. **(D)** PF74 time-of-addition based measurements of the nuclear import kinetics for SiR-tetrazine-labeled WT and CA* pseudoviruses. Here, 2.5 μM PF74 was added to cells at 0, 2, 4, 8, 10, and 24 hpi (*T*_50_ for CA* = 6.27 hpi, *T*_50_ for WT CA = 6.58 hpi, mean and SD shown for three different viral preps). *T*_50_ values for CA^WT^ and CA* were not statistically different (*p* = 0.7, unpaired *t* test). **(E)** TRIMCyp restriction assay to assess the cyclophilin A binding capabilities of SiR-tetrazine-labeled CA* pseudovirions. TZM-bl cells expressing TRIMCyp were infected with CA*/untagged G89V pseudoviruses, and the resulting luciferase signal was measured after 48 h. Firefly luciferase light units were normalized to p24 ELISA content, and the CA*/untagged G89V ratio was normalized to 100% G89V infectivity (CA*:G89V: *p* = 0.0008, CA^WT^:G89V: *p* = 0.01, CA*:CA^WT^: *p* = 0.32, CA^WT^:G89V: *p* = 0.003, unpaired *t* test, mean and SD shown for two different viral preparations). **(F)** Kinetics core uncoating measured *in vitro.* CA* and WT pseudoviruses were lysed with 100 μg/mL saponin and fixed/immunostained with anti-CA (AG3.0) antibody. Data are from two independent preps, mean and SD plotted. **(G)** Kinetics of *in vitro* uncoating of HIV-1 pseudoviruses containing WT CA*/untagged WT CA and K203A CA*/untagged K203A. A fraction of particles retaining above-threshold CA* signals are responsible for a nonzero plateau. Rate constants (*k*) for WT and K203A CA* pseudoviruses are 0.08 and 1.05, respectively. Data are from two independent viral preps, mean and SD are plotted. Immature cores were excluded from analysis.

I91 amber mutant pseudoviruses allowed for direct virus labeling by incorporating TCO^51,52^ in the context of the NL4–3ΔEnv and pR9ΔEnv HIV-1 clones^6,26,30,49^ through strain-promoted inverse electron-demand Diels–Alder cycloaddition click-labeling^34,52,53,55,58^ of CA with tetrazine-conjugated fluorophores (hereafter referred to as CA*, Figure 1A). Infectivity of pseudoviruses containing pure I91 CA* was markedly reduced, while complementation with CA^WT^ rescued infectivity (Figure S2B). Specific infectivity of pseudoviruses produced using the 1.5:1 I91 mutant/WT plasmid ratio and labeled with Silicon Rhodamine-tetrazine (SiR-tetrazine) was impacted less than 2-fold (*p* = 0.055, Figure 1B).

To assess the extent of incorporation and labeling of CA* in the context of mixed CA*/CA^WT^ pseudoviruses, virions were adhered to coverslips, labeled with SiR-tetrazine, fixed, permeabilized and immunostained for p24/CA using the AG3.0 p24 antibody that preferentially recognizes mature capsid.^59^ Using this protocol, 58.9% of CA*-labeled cores colocalized with mature p24/CA foci (Figure 1D). The less-than-optimal colocalization of CA* with p24/CA may be due to the use of cotransfection with 5 plasmids, low efficiency of amber codon suppression and/or incomplete click reaction with HIV-1 core-incorporated CA* (see below for additional analyses/validation). Specificity of CA* labeling is evident from the lack of discernible SiR puncta or background signal, outside of p24 immunostained cores that contain WT CA (Figure S3A). Although recent reports suggest that HIV-1 backbone possesses amber codons in Vif, Vpu, and Rev,^60^ the lack of off-target staining of the WT CA vector in our experiments (Figure S3A) likely reflects the absence of these proteins in budding virions.

To determine the optimal conditions for CA* pseudovirus labeling with tetrazine dyes, we varied the time of labeling with 250 nM SiR-tetrazine. Quantifying single virus CA* intensity distributions over time demonstrated that the half-time for efficient tetrazine labeling is 6.2 min (Figure S3B) and that our standard 20 min tetrazine labeling achieves ∼77% of CA* labeling *in vitro*.

Whereas a single HIV-1 particle, on average, contains several thousand Gag polyproteins (>2500),^61,62^ only about 1500 CA monomers are capsid lattice-associated in mature virions.^61−63^ It has been reported that permeabilization of the viral envelope results in release of a portion of labeled CA that was not incorporated into the capsid lattice, trapped within the viral membrane.^25,32,44^ We therefore asked if the intraviral distribution of CA* was unaffected by the mutation and click labeling. In general agreement with the published results, exposure of mixed CA*/CA^WT^ pseudoviruses to membrane-permeabilizing saponin released roughly half of the virus’ CA* signal (Figure S3C, D).

Since amber codon suppression is not very efficient in mammalian cells, the actual CA* and CA^WT^ ratio is likely to be lower than the respective plasmid ratio. To estimate the actual ratio of CA* and CA^WT^ in virus producing cells, HEK293*T*/17 cells were transfected with CA* and CA^WT^ viral vectors using a plasmid ratio of 1.5:1, and the resulting ratio of expressed proteins was determined by Western blotting. To ease the assessment of CA*/WT CA ratio, transfections were carried out in the presence of 200 nM of the HIV-1 protease inhibitor, Saquinavir (SQV)^64^ to block Gag processing and simplify ratiometric analysis of a single Gag band. Analysis of the Gag band intensities demonstrates that there is ∼1.3 times more CA^WT^ produced compared to CA* for the 1:1.5 plasmid ratio (Figure S5). Thus, the amber suppression efficiency reduces the TCO-tagged Gag expression in HEK/293T cells relative to untagged Gag by ∼2.0-fold. The less than 2:1 ratio of unlabeled to labeled CA that rescues HIV-1 infectivity is in sharp contrast to the GFP-tagged CA, which requires 10–15-fold excess of untagged CA to produce infections viruses.^25,32^

To test if transport of SiR-tetrazine-labeled CA* cores through the nuclear pore is impaired, we analyzed the nuclear import kinetics by performing a PF74 time-of-addition assay.^26,41,65,66^ At low concentrations (≤2.5 μM), PF74 blocks nuclear import, likely by stabilizing HIV-1 cores, whereas high concentrations (≥10 μM) of this compound impair reverse transcription and displace imported viral complexes from nuclear speckles.^6,26,41,65,67^ Thus, the time course of virus escape from a low dose of PF74 reflects the kinetics of HIV-1 nuclear import.^26^ These experiments revealed that the kinetics of nuclear import of mixed CA*/CA^WT^ pseudoviruses is indistinguishable from that of control pseudoviruses (Figure 1D).

The CypA binding loop of CA interacts with CypA, and likely with Nup358, and plays an important role in HIV-1 infection through modulating capsid interactions with a multitude of host factors (including restriction factors).^5,8,68−71^ We therefore asked if the mixed cores containing CA* retain the ability to functionally interact with the owl-monkey TRIMCyp, which binds to the CypA binding loop and restricts infection by prematurely degrading the capsid lattice.^19,21,44,72−74^ Mixed CA* pseudoviruses were produced by coexpressing the untagged G89V CA mutant within the CypA binding loop, which is resistant to TRIMCyp restriction.^44,75^ Having G89V CA present in the mixed capsid lattice allows for probing TRIMCyp binding to CA* (or CA^WT^ in control samples), using an indirect infectivity readout.^19,21,44^ CA*/CA^G89V^ and CA^WT^/CA^G89V^ viruses were equally sensitive to TRIMCyp restriction, whereas control viruses containing only pure G89V CA and CA^WT^ cores were, respectively, resistant and sensitive (10-fold reduction in infection) to this factor (Figure 1F). This difference in restriction between mixed G89V/CA^WT^ cores and pure CA^WT^ cores may be due to inefficient incorporation of CA^WT^ into CA^G89V^ containing viral capsids, consistent with imperfect p24/CA* colocalization shown in Figure 1C. These results suggest that CA labeling at the position I91 does not impair CypA binding to HIV-1 cores containing a mixture of CA^WT^ and CA* and that CA* is incorporated into the capsid lattice, with the CypA binding loop exposed to cytosolic factors. These data agree with the previous study^57^ reporting that the I91 mutation does not affect CypA binding to the capsid lattice.

To ensure that our CA* labeling approach does not impact capsid stability, we compared the stability of CA*/WT and WT cores using an *in vitro* uncoating assay. WT HIV-1 uncoating was measured by immunostaining for p24/CA. Briefly, we permeabilized coverslip-attached pseudoviruses with saponin, fixed the exposed cores at 0, 5, 15, and 30 min after lysis, and immunostained with the anti-HIV-1 p24 AG3.0 antibody which detects mature cores.^59,76^ There was no significant difference in *in vitro* uncoating kinetics (loss of p24 foci) between mixed CA*/WT and unlabeled WT mature cores (Figure 1F). This suggests that our CA* labeling protocol does not majorly impact core stability *in vitro* and, therefore, justifies the use of these mixed cores for uncoating experiments in a cellular context. Accordingly, time-resolved imaging of single mixed WT/CA* core uncoating revealed a similar kinetic of loss of CA*-labeled cores after saponin lysis (Figure 1G). Immature cores exhibit constant CA* fluorescence upon membrane lysis, whereas mature cores lose a membrane-trapped fraction of CA* after saponin addition. Of note, ∼29% of mature cores retained postlysis levels of CA* fluorescence within 30 min of saponin addition at room temperature, likely representing “closed” capsids^48^ that failed to uncoat under these conditions. In contrast, markedly accelerated uncoating was observed for K203A CA* mutant capsids known to form highly unstable cores that rapidly disassemble after viral fusion or lysis (Figure 1H and Figure S4A).^77^ Interestingly, a small fraction of the K203A CA*/untagged K203A mutant cores (∼10%) exhibited delayed uncoating over the course of 30 min (Figure S4B). Approximately 15% of mature particles retained very weak but detectable levels of CA* fluorescence by 30 min after saponin lysis (Figure S4C). Thus, loss of WT/CA* signal over the time course postlysis is primarily caused by the capsid lattice dissociation, whereas residual fluorescence retained by a minor fraction of unstable cores after uncoating may correspond to vRNP-associated CA*.^25,32,44^

Together, these results demonstrate that our direct CA-labeling approach using mixed Mutant/WT CA cores does not majorly affect the core stability or virus functionality, making it suitable for visualizing the sites and the kinetics of uncoating in target cells.

### Colabeling of HIV-1 Capsid with CA* and Core-Entrapped Fluid Phase Marker Reveals Two-Step Uncoating of Cell-Free Viruses

**Figure 2:**
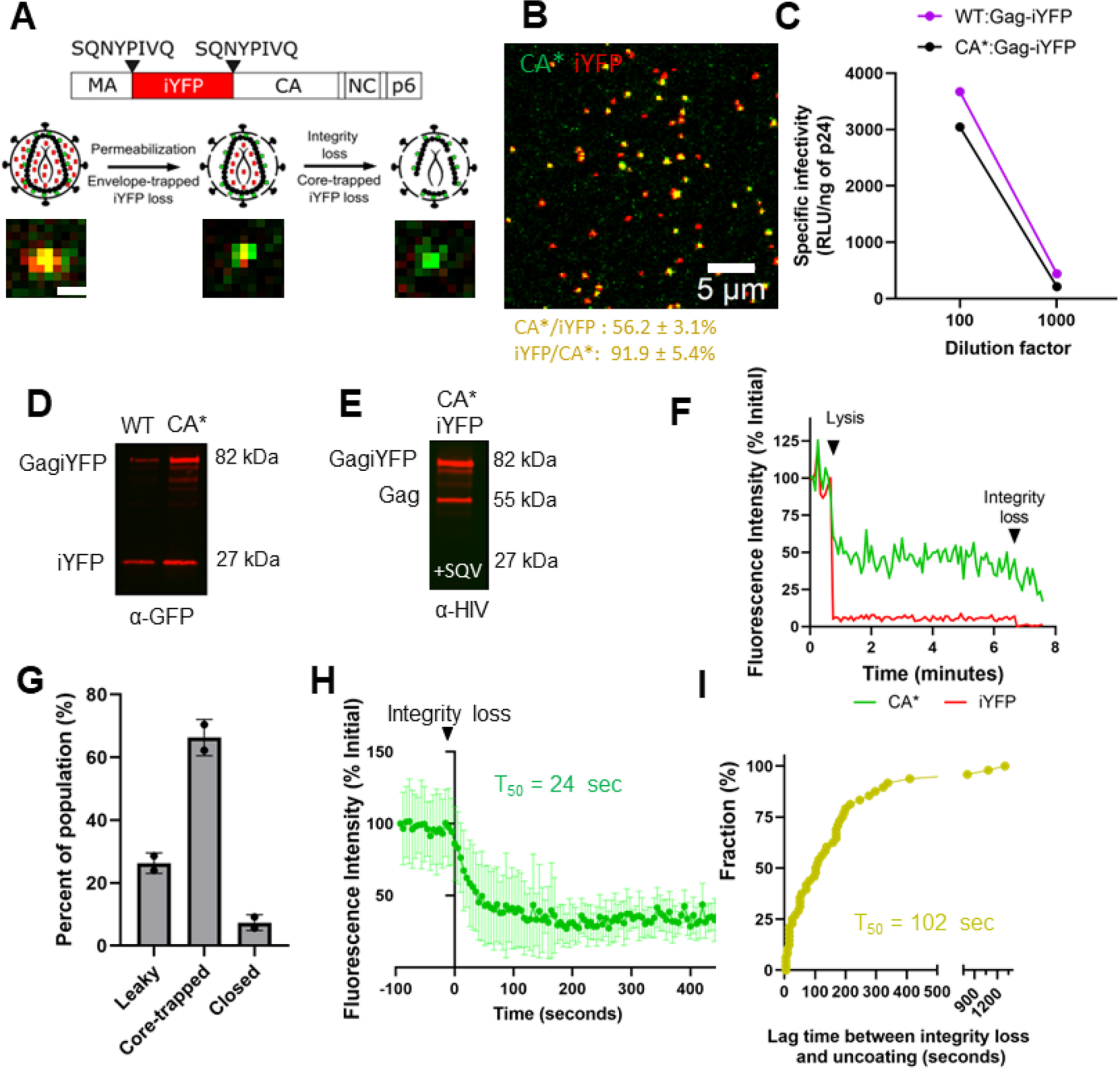
Multistep uncoating of CA*:Gag-iYFP pseudovirus cores *in vitro*. (A) Schematic for CA*:Gag-iYFP pseudovirus labeling with the fluid phase marker iYFP for assessing capsid integrity. Schematics and images of cell-free CA*:Gag-iYFP particle integrity loss and uncoating are shown (scale bar is 1 μm). **(B)** Representative image of cell-free CA*:Gag-iYFP pseudoviruses labeled with SiR-tetrazine. Percent colocalization is listed below in the yellow font, mean and SD are plotted. **(C)** Representative plots for specific infectivity of control (WT) and SiR-tetrazine-labeled CA*:Gag-iYFP pseudoviruses in TZM-bl cells measured as firefly luciferase signal (RLU) and normalized to p24 content. **(D)** Western blot displaying GagiYFP cleavage in WT:iYFP and CA*:Gag-iYFP lysates (50 pg of virus p24 loaded). Anti-GFP living colors antibody was used for iYFP detection. Estimated protein molecular weights (kDa) listed next to bands. We did visualize a somewhat higher uncleaved GagiYFP precursor bands (∼82 kDa) for CA*:Gag-iYFP pseudoviruses, but this is likely due to the reduced efficiency of CA* translation, allowing for more GagiYFP incorporation during virus assembly than WT. There were additional MA-iYFP-CA bands (synonymous with p41 MA-CA band) on the blot, but these are likely due to a small fraction of immature particles present in the population. **(E)** Estimation of CA*:Gag-iYFP ratio in HIV-1 cores. Producer cells were treated with 200 nM saquinavir to block virus maturation; anti-HIV serum antibodies were used for virus detection (50 μg of p24 loaded). Estimated protein molecular weights (kDa) listed next to bands. Viruses were produced in the presence of 200 nM saquinavir to block maturation and ease densitometry analysis, as described in Figure S1C. **(F)** Representative intensity trace for two-phase iYFP release phenotype from CA*:Gag-iYFP cores. Arrows and identifying text signify time points of pseudovirus lysis and integrity loss. **(G)** Quantification of Gag-iYFP release phenotypes from CA*:Gag-iYFP pseudoviruses *in vitro*. Each population is normalized to the total particle population. **(H)** Ensemble CA* intensity trace plot of coverslip immobilized CA*:Gag-iYFP pseudovirus CA* fluorescence intensity after iYFP loss. Traces were aligned at iYFP loss (*t* = 0) and normalized to the CA* signal at this time point. Averaged CA* traces were terminated at the time of complete loss of CA* signal, with only above background values plotted. **(I)** The lag times between integrity loss and terminal uncoating from the particles used in Panel H. Kinetics curve is normalized to the final time point. Kinetics curve fitted with single exponential equation.

Having validated our CA* labeling approach, we sought to resolve early steps of HIV-1 capsid uncoating preceding the terminal loss of CA*. Single capsid integrity loss has been visualized through incorporation of a fluid phase GFP marker into viral cores using the GagiGFP construct.^24,48,78^ This Gag construct containing “internal” GFP (iGFP) generates a fluid phase iGFP marker upon virus maturation.^24,27,48,79^ We swapped GFP for YFP in the GagiGFP backbone^47^ to make a Gag-iYFP-Pol construct (hereafter referred to as Gag-iYFP).^24,27,47,48^ iYFP trapped inside an intact HIV-1 core is released upon the formation of small defects (>4 nm),^80^ thus acting as a core integrity marker (Figure 2A).^24,27,48^

By producing CA*:Gag-iYFP pseudoviruses similarly to the single-labeled CA* virions, we achieved 56.2% colocalization of iYFP with CA*, but 91.9% of CA* signal was associated with iYFP (Figure 2B). Thus, the overwhelming majority of CA* cores contain a fluid phase fluorescent marker. This double-labeling approach did not considerably reduce specific infectivity (*p* = 0.02, Figure 2C) compared to CA^WT^:Gag-iYFP pseudoviruses. Immunoblotting revealed cleavage of GagiYFP in double-labeled pseudoviruses, as evidenced by a processed iYFP band (∼27 kDa, Figure 2D). Similar to the ratio of CA*/CA^WT^ in single-labeled pseudoviruses, transfections of producer cells with a 1.5:1 plasmid ratio (CA*:Gag-iYFP, see Methods) yielded approximately 1.6-fold more GagiYFP to CA* (Figure 2E).

We examined disassembly of CA*:Gag-iYFP cores that contained releasable iYFP by permeabilizing HIV-1 pseudoviruses adhered to coverslips with saponin, as above. Imaging the resulting loss of iYFP and CA* signals revealed three distinct types of iYFP release events: (1) immediate and complete loss of iYFP upon viral membrane permeabilization, likely corresponding to “leaky” cores, (2) a two-phase loss of iYFP: initial release of most of the viral iYFP immediately after addition of saponin, followed by a second loss due to defect formation in intact cores,^24,27,48^ and (3) initial iYFP loss upon saponin addition without loss of core-entrapped iYFP, likely representing “closed” cores that failed to uncoat by the end of an imaging experiment.^48^ A representative single particle trace of two-phase release is depicted in Figure 2F. Examples of leaky and closed traces are shown in Figure S6A, B. We found that 66.3% of CA*:Gag-iYFP cores exhibit a two-phase iYFP loss, whereas only a minor fraction of cores is “leaky” (26.3%) or “closed” (7.4%) (Figure 2G, n = 505). Thus, the majority of HIV-1 cores colabeled with CA* and iYFP appears to contain intact capsid lattice. It is possible that partially processed Gag-iYFP may be responsible for the residual iYFP fluorescence after loss of capsid integrity. Interestingly, we often observe gradual loss of CA* content after loss of iYFP, as shown in Figure 2F, suggesting that uncoating *in vitro* may occur through a gradual loss of CA, rather than sudden disassembly, which was suggested by an *in vitro* uncoating assay using indirect capsid labeling.^48^

To determine if the fluid phase marker, iYFP, can be released on demand from the CA*:Gag-iYFP labeled cores, coverslip-adhered pseudovirus membrane was permeabilized by saponin in the presence of PF74, which, at higher concentrations, induces core integrity loss but stabilizes the capsid lattice.^25,27,41,48,67,81^ At 10 μM, PF74 induces a rapid and complete loss of iYFP from CA* cores, except for 12.8% of mature cores retaining iYFP (Figure S7). In spite of inducing defect formation, this concentration of drug markedly stabilizes the CA* signal (Figure S7), in agreement with a previous study.^48^ It is possible that the remaining 12.8% of cores may contain a partially processed GagiYFP that is not releasable upon lysis. It is also possible that these cores may be more resistant to PF74-mediated integrity loss and require >30 min to release iYFP. We can thus utilize PF74 to promote defects in CA*:Gag-iYFP capsids in the cells in order to ascertain the intactness of these HIV-1 cores.

To investigate whether lattice defect formation leading to iYFP release is associated with detectable loss of CA*, we plotted an ensemble average of 49 single particle intensity traces aligned at the time of the second iYFP release step. Most (∼82%) cores do not exhibit a detectable reduction in the CA* signal at the point of iYFP loss, but CA* intensity gradually decays after that point with a half-time (*T*_50_) of 24 s (Figure 2H). Interestingly, ensemble average of CA* intensities aligned at the time of iYFP loss suggests that surviving cores tend to retain, on average, ∼33% of the initial CA* fluorescence signal for some time before terminal uncoating (Figure 2H and S8A). A heat map of overlaid individual CA* intensity traces (Figure S8B) depicts gradual CA* loss after defect formation with some cores exhibiting residual CA* fluorescence after iYFP loss. The lag time between integrity loss and terminal uncoating for all 49 CA*:Gag-iYFP cores in Figure 2H has a *T*_50_ of 102 s (Figure 2I). These results suggest that, upon defect formation, the capsid gradually loses CA* until terminal disassembly. The residual ∼33% CA* observed may represent a metastable state preceding terminal uncoating, consistent with the published results.^48,82^ Importantly, because most cores did not exhibit detectable CA* loss upon loss of capsid integrity, the capsid lattice defect allowing iYFP release *in vitro* is likely to be relatively small.

### HIV-1 Uncoating in the Cytosol and at the Nuclear Pore Occurs in Two Steps

**Figure 3:**
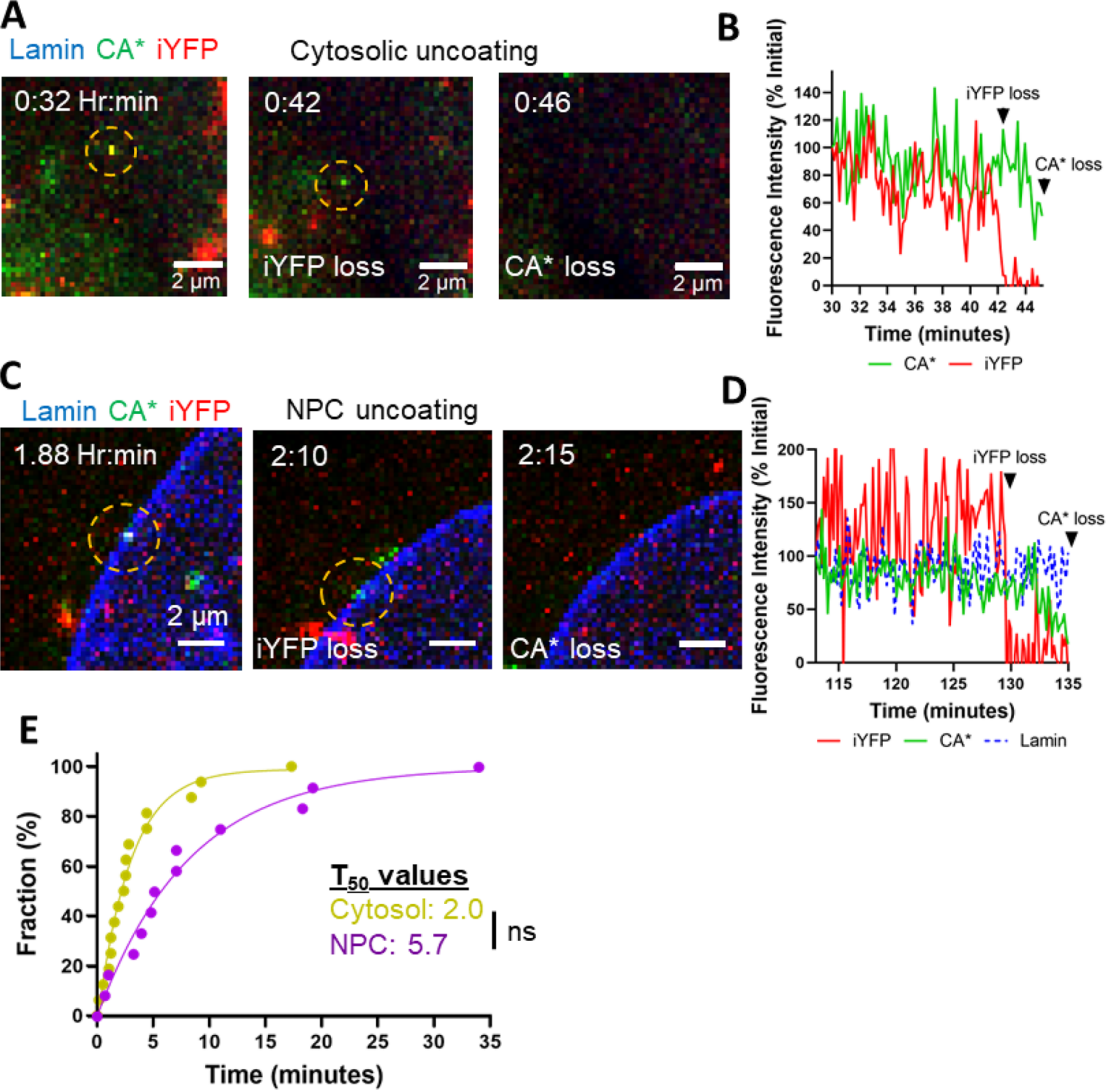
**Two-step uncoating exhibited by cytosolic and NPC-localized cores. (A)** Live-cell micrograph for a representative two-step cytosolic CA*:Gag-iYFP uncoating event. Yellow dashed circle highlights the core of interest. White text highlights times points for integrity loss and uncoating. Large CA*:Gag-iYFP foci within the cytosol are likely nonfused pseudoviruses trapped in endosomes (refer to Figures 2A,B & S3B,C). Temporal resolution is ∼10 s per frame. **(B)** Single particle intensity trace of cytosolic uncoating event from panel A. **(C)** Live-cell micrograph for a representative two-step NPC-localized CA*:Gag-iYFP uncoating event. Yellow dashed circle highlights particle of interest with white text describing uncoating event. Temporal resolution is ∼10 s per frame. **(D)** Single particle intensity trace of CA*:GagiYFP core from panel C. **(E)** Two-step uncoating lag-time kinetics for cytosolic, NPC-docked, and nuclear uncoating events. *T*_50_ values for each uncoating position are listed beside the graph. Statistical analysis of data was performed with Mann–Whitney rank sum test, *p* = 0.07.

We took advantage of our double-labeled HIV-1 capsids to assess the sites of capsid uncoating in GHOST(3) cells.^83^ These cells were utilized for HIV-1 uncoating analyses due to their low background staining with SiR-tetrazine compared to HeLa-derived cells and thin nucleus, which facilitates 3D time-lapse imaging. We made GHOST(3) cells stably expressing SNAP-Lamin B1^26,44,84^ (referred to as GHOST-SNAP-lamin cells) to visualize the nuclear membrane by staining with SNAP-reactive dye. We visualized uncoating in the cytoplasm (n = 17) and at the nuclear pore complex (NPC, n = 13). In these experiments, click-labeling of CA*:Gag-iYFP cores was carried out immediately after virus-cell binding, as described in the Methods section, and imaging started at 30 min postinfection. We detected two-step uncoating (iYFP loss preceding terminal CA* loss) of CA*:Gag-iYFP labeled cores in the cytosol and at NPC (Figure 3A-D, Movies S1–S2). The lag time between iYFP loss and terminal CA* loss is relatively short, with *T*_50_ = 2 min for cytosolic cores and *T*_50_ = 5.7 min for NPC-localized cores (Figure 3E). Although the lag between the steps of uncoating is somewhat longer for NPC-docked cores, the difference is not statistically significant (*p* = 0.07).

### No Detectable Loss of CA* or iYFP Occurs upon HIV-1 Nuclear Import

**Figure 4:**
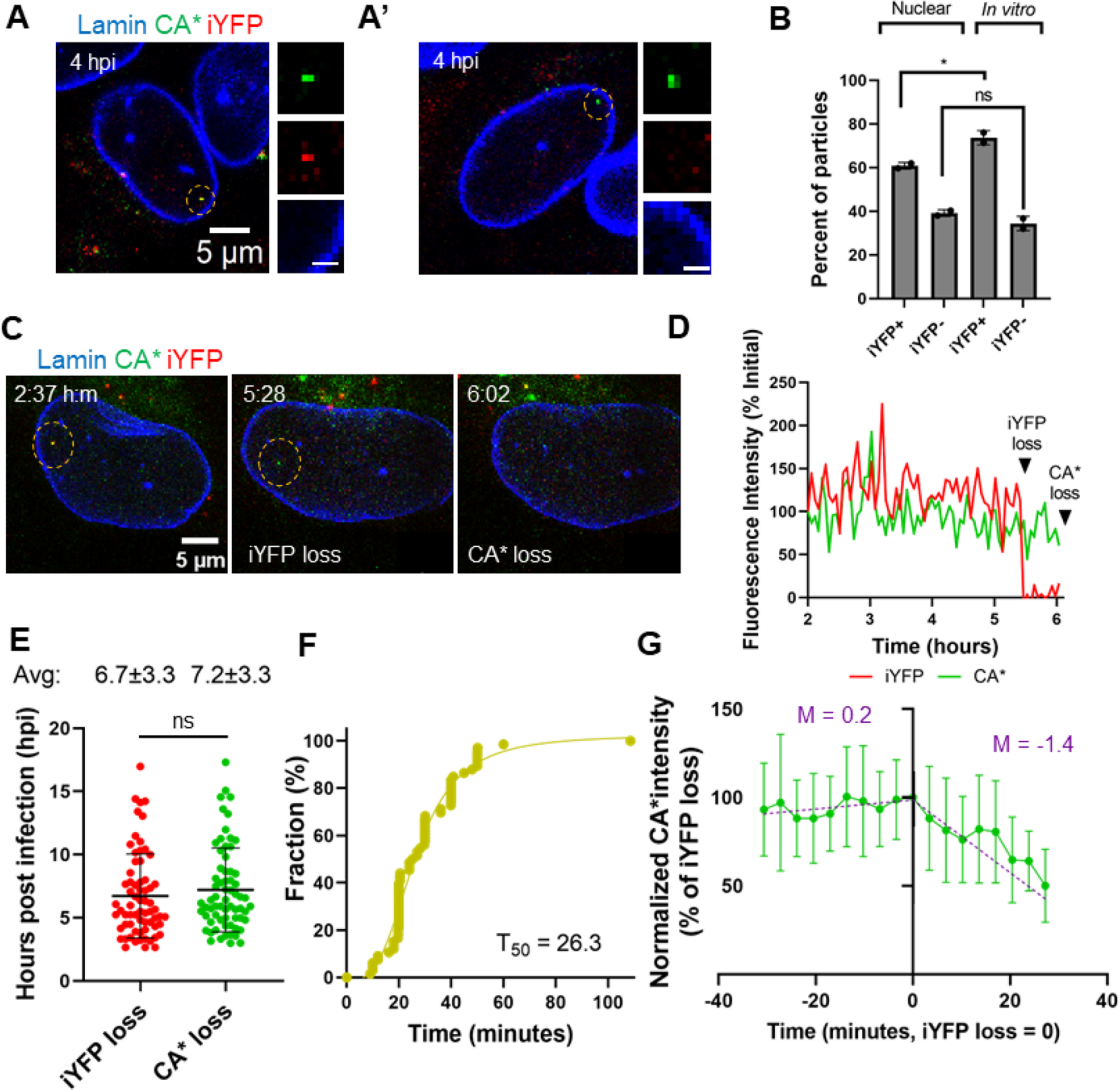
**Intact cores enter the nucleus of GHOST-SNAP-lamin cells and undergo sequential loss of capsid integrity and terminal uncoating. (A)** A representative image showing a nuclear CA*:Gag-iYFP capsid. (A′) A representative image of a nuclear capsid lacking iYFP signal. Dashed yellow circles mark the particles of interest. Zoomed in images of the nuclear CA*:Gag-iYFP cores are shown to the right. Scale bar is 1 μm. **(B)** Quantification of the fractions of YFP- and YFP+ CA* cores in nuclei and inside saponin lysed viral particles attached to coverslips. Data were normalized to the total number of nuclear particles. Coverslip-attached particles were quantified by subtracting the population of leaky CA*:Gag-iYFP cores from core-trapped and closed iYFP species (see Figure 2G). For in vitro cores, iYFP+ corresponds to all CA* foci positive for core-trapped iYFP, and iYFP- represents leaky particles along with CA* foci that were not copackaged with Gag-iYFP during production. Means and SD are plotted from two independent experiments. Significance derived from unpaired *t* test (*p* = 0.03 for iYFP+, 0.2 for iYFP-). **(C)** Representative live-cell micrograph of CA*:Gag-iYFP integrity loss and uncoating in the nucleus of an infected GHOST-SNAP-lamin cell. Particle of interest is highlighted by the yellow dashed circle. Integrity loss and uncoating are marked by white text within the image. Temporal resolution is ∼3 min per frame. **(D)** Representative single particle intensity traces for the CA*:Gag-iYFP core from panel F. Integrity loss and uncoating are marked by arrows. **(E)** Waiting times of all CA*:Gag-iYFP integrity loss and uncoating events within the nucleus. Mean and SD are plotted. Average integrity loss and uncoating times are displayed above each column with standard deviations. **(F)** Cumulative distributions of the lag time between integrity loss and uncoating of single particles. Mean and SD are plotted. **(G)** Ensemble intensity trace plot demonstrating CA* uncoating dynamics after iYFP loss. Purple text lists the slope for the per-integrity loss and post integrity loss ensemble average for the CA* intensity trace. Traces longer than 30 min were cut off for consistency.

Having demonstrated the ability of the CA*:Gag-iYFP virus to report distinct integrity loss and uncoating events *in vitro* and in the cytoplasm/nuclear envelope, we asked if intact capsids (defined as CA* labeled cores containing releasable iYFP) can enter the cell nucleus. To test this, GHOST-SNAP-lamin cells were infected with CA*:Gag-iYFP labeled pseudoviruses and fixed at 4 hpi to determine the amounts of double-labeled and CA* only labeled cores in the nucleus. Over half (60.8%) of the nuclear CA* foci were iYFP positive, suggesting that intact capsids can enter the nucleus of infected cells (Figure 4A). The nuclear CA* cores lacking iYFP (Figure 4A’) may, in principle, arise from loss of integrity, either before nuclear entry or in the nucleus. Alternatively, these could be intact cores that were not labeled with iYFP upon production (Figure 2B). Interestingly, the fraction of iYFP-retaining cores after mild lysis of coverslip-adhered CA*:Gag-iYFP pseudoviruses was close to the fraction of iYFP-positive nuclear cores (Figure 4B), supporting the notion that the capsid can enter the nucleus without losing its iYFP content.

To further test if nuclear CA* foci represent intact/mostly intact capsid structures, we measured the fluorescence intensity distribution of free CA* cores in the cytosol, nuclear membrane, and nucleus. To differentiate intact virions trapped in endosomes, which contain approximately twice as much CA* as postfusion/lysis cores (Figure S3C and^25,34,61^), from free cytosolic cores, the cells were infected in the presence of the fixable membrane marker mCLING-Atto488.^85^ mCLING-Atto488 associates with both the viral and cell membranes^31,38^ (see also Figure S9A), and, thus, colocalizes with CA* signal from intact virions (Figure S9B, yellow circles), which were excluded from analysis, but not with postfusion cores in the cytoplasm. Quantification of the intensities of free CA* cores (Figure S9B, white arrows) in the cytoplasm, at the nuclear membrane, and in the nucleus of fixed cells revealed no significant difference in intensity distributions (Figure S9C), suggesting there is no detectable loss of capsid protein upon HIV-1 nuclear import.

To extend the analysis of CA* intensity in the cytoplasm and nucleus of fixed cells, we imaged entry of individual CA* cores into the nucleus of living GHOST-SNAP-lamin cells. Importantly, single virus tracking did not detect loss of fluorescence intensity upon nuclear import of CA* cores (n = 8, Figure S9D and Movie S3). Individual traces for each nuclear import event demonstrate no discernible loss of fluorescence intensity for individual CA* intensity traces (Figure S10) and in the ensemble average plot across all cores aligned at the time of nuclear import (Figure S9E). Unfortunately, we were unable to resolve cytosolic cores docking at the NPC in GHOST-SNAP lamin cells. Although the relatively quick virus docking and nuclear entry in these cells precluded the analysis of CA* intensity prior to and at the time of docking, our fixed cell data support that there is no detectable loss of CA* occurs during docking and passage through the NPC. Collectively, these data suggest that HIV-1 cores with largely intact capsid lattices may enter the nucleus of GHOST(3) cells.

### Terminal CA* Loss from Nuclear HIV-1 Capsids Is Preceded by a Loss of Core-Trapped iYFP

We next asked if nuclear cores lose iYFP and CA* signals and if release of iYFP is temporally linked to terminal loss of CA*. To visualize capsid integrity loss and uncoating, we tracked 67 CA*:Gag-iYFP-labeled pseudoviruses in live GHOST-SNAP-lamin cells between ∼1.5 and ∼22 hpi. Representative single particle images and fluorescence traces show iYFP release followed by terminal loss of CA*, which are interpreted as loss of integrity followed by terminal uncoating (Figure 4C, D, and Movie S4). Importantly, for all visualized nuclear cores colabeled with CA*:Gag-iYFP, loss of core integrity culminates in terminal uncoating in a two-step manner. No instances of simultaneous loss iYFP and CA* were observed. The average time for capsid integrity loss was 6.7 hpi, whereas subsequent terminal uncoating occurred at 7.2 hpi (Figure 4E). The average lag time between iYFP release and terminal loss of CA* in GHOST-SNAP-lamin cells is 29 min (Figure 4F), suggesting that loss of integrity of the capsid lattice may be an intermediate step prior to terminal uncoating in the nucleus. Interestingly, two-step uncoating in the nucleus appears to occur >5-fold slower than uncoating events within the cytosol or at the NPC, suggesting that nuclear host factors stabilize the capsid lattice. No detectable loss of CA* is associated with iYFP release, as evidenced by ensemble averaged CA* intensity traces (n = 18) aligned at the time of integrity loss (see also individual CA* intensity traces, Figure S11). This indicates that the initial defect responsible for iYFP release is not associated with a significant loss of CA from the lattice. While no instant loss of CA* at the time of iYFP release is detected, the CA* signal appears to gradually decay from that point on with a slope of 1.4%/min (Figure 4G). A gradual loss of CA* following an initial lattice defect formation is consistent with our *in vitro* uncoating results (Figure 2H), except that gradual uncoating in the nucleus is much slower compared to *in vitro* uncoating (*T*_50_ = 24 s *in vitro vs* 36 min (1.4%/min loss of CA*) in nucleus).

**Figure 5:**
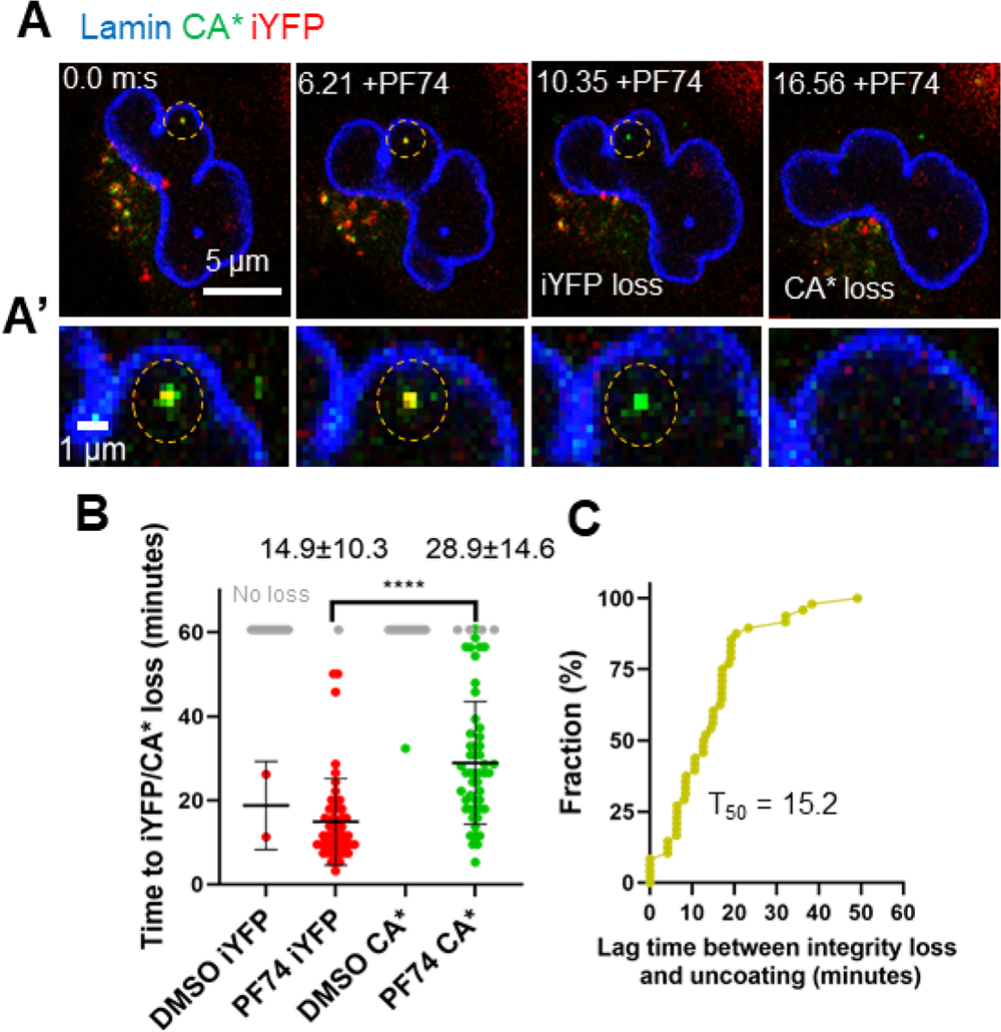
PF74-induced iYFP loss and CA* degradation in the nucleus of GHOST-Lamin cells. (A) Live-cell micrograph of a nuclear CA*:Gag-iYFP core in GHOST-SNAP-lamin cell treated with 10 μM PF74. The particle of interest is marked with the yellow dashed circle. **(A′)** Zoomed in section of the live-cell micrograph from panel C. **(B)** The kinetics of iYFP and CA* loss in PF74 and DMSO treated GHOST-SNAP-lamin cells (*p* < 0.0001, Mann–Whitney rank sum test). Gray dots represent CA*:Gag-iYFP particles that remain after 1 h of imaging. Mean and SD are plotted. Average time and SD are listed above each PF74 column. **(C)** Cumulative distribution plot for the lag time between integrity loss and PF74-induced capsid degradation in the nucleus of cells.

To ensure that the nuclear cores contain a releasable pool of iYFP, we induced the release of iYFP by treating infected GHOST-SNAP-lamin cells with PF74, similar to *in vitro* experiments (Figure S6). Addition of 10 μM PF74 to cells at 3 hpi, induced a loss of iYFP from CA* cores, while the CA* signal was retained for an additional several minutes (Figure 5A, A’, and Movie S5). The majority (88.5%) of iYFP-positive CA* cores released iYFP upon PF74 addition, while retaining CA* signal. Only 1.6% of CA*:Gag-iYFP cores were not sensitive to PF74 treatment, with no observable loss of iYFP or CA* during the 1-h incubation with PF74. In contrast, there were only two spontaneous integrity loss events and one CA* loss event that was visualized during this imaging window in control DMSO treated cells, showing that PF74 potently triggers integrity loss in nuclear HIV-1 cores. We did detect several instances (9.9%) of iYFP and CA* being lost within the same image frame, but this is likely due to our limited temporal resolution (∼2 min) that is insufficient to resolve short lags between the two events. By observing 50 nuclear double-labeled cores, we find that the average time for iYFP loss after PF74 treatment is 14.9 min, followed by CA* loss after a significant lag of 13.9 min, on average (Figure 5B, C). Interestingly, nuclear CA* cores become much more mobile upon PF74 treatment, consistent with our previous work reporting increased mobility of nuclear cores displaced from nuclear speckles and subsequent degradation after PF74 treatment.^6^ Thus, delayed loss of CA* signal after release of iYFP upon PF74 addition can be attributed to potential degradation of HIV-1 lattice displaced from nuclear speckles.^6^

### Reverse Transcription Does Not Majorly Impact the Lag between Two Uncoating Steps in the Nucleus

**Figure 6:**
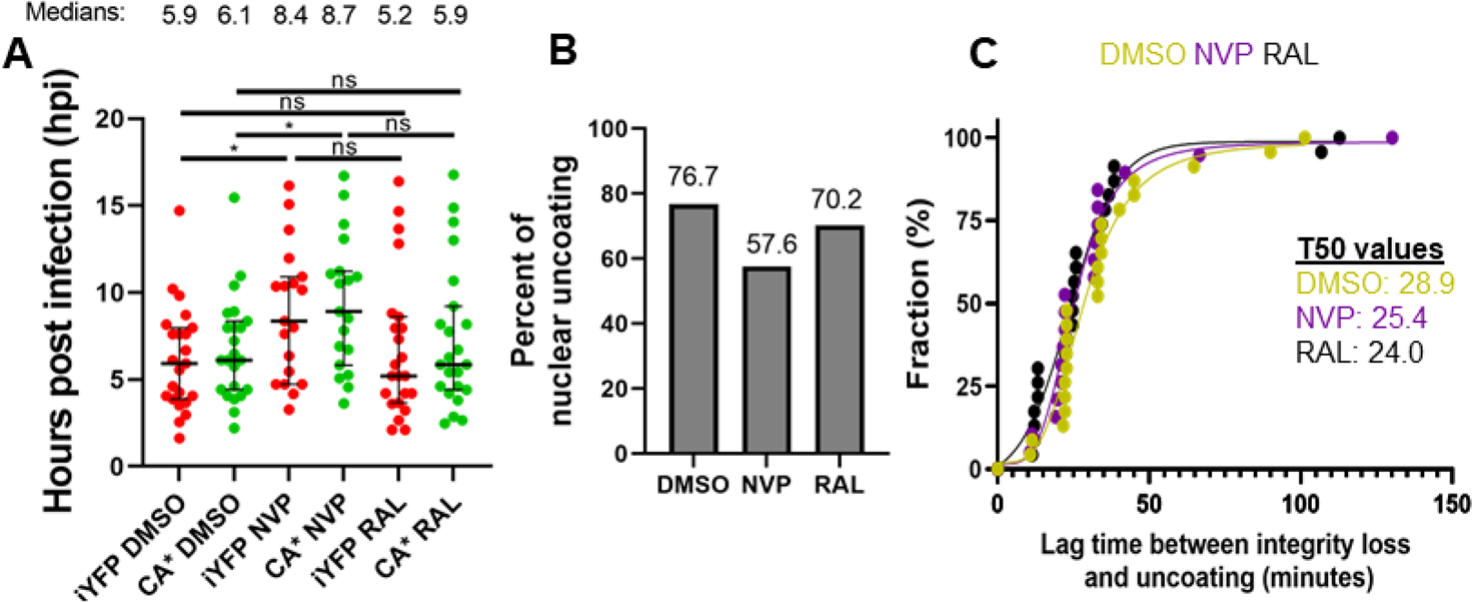
**Reverse transcription accelerates capsid integrity loss but does not affect the lag time between integrity loss and uncoating. (A)** Waiting times for all CA*:Gag-iYFP integrity loss and uncoating events with DMSO, 10 μM NVP, and 10 μM RAL treatment. Median and interquartile range are plotted. Average integrity loss and uncoating times are displayed above each column with standard deviations. Temporal resolution is ∼11 min per frame. **(B)** Uncoating efficiency of DMSO and NVP treated cells, with 19.1% reduction in NVP treated CA*iYFP cores, with minimal effect with RAL present. **(C)** Cumulative distributions of the integrity loss and terminal uncoating lag times in DMSO, NVP, and RAL treated cells. Statistical analysis of data performed with Mann–Whitney rank sum test.

We next asked if reverse transcription influences the lag time between integrity loss and uncoating within the nucleus. Toward this goal, the kinetics and efficiency of integrity loss (iYFP release) and uncoating (CA* loss) in GHOST-SNAP-lamin cells in the presence of the reverse transcriptase inhibitor NVP (10 μM) was measured. The integrase strand-transfer inhibitor, Raltegravir (RAL, 10 μM), was used as a control since integration occurs after capsid disassembly. Inhibition of reverse transcription delayed both the capsid integrity loss and uncoating by ∼2.5 h each. The average time for integrity loss and uncoating in the presence of DMSO is 6.2 and 6.8 hpi, as compared to 8.8 and 9.3 hpi, respectively, in NVP treated cells (Figure 6A). NVP treatment also causes a 19.1% decrease in the probability of CA* loss observed within our imaging window (Figure 6B), suggesting that reverse transcription plays a role in the uncoating process, in agreement with.^2,19,25,33,36,39,41,67,86,87^ Interestingly, we do not observe significant differences between the two-step uncoating lag times for CA*:Gag-iYFP cores treated with DMSO, NVP, and RAL (Figure 6C). This indicates that reverse transcription plays a role in initiating capsid integrity loss but may not have a major role in the terminal uncoating process after integrity loss.

### HIV-1 Undergoes Two-Step Uncoating in Different Cell Types

**Figure 7:**
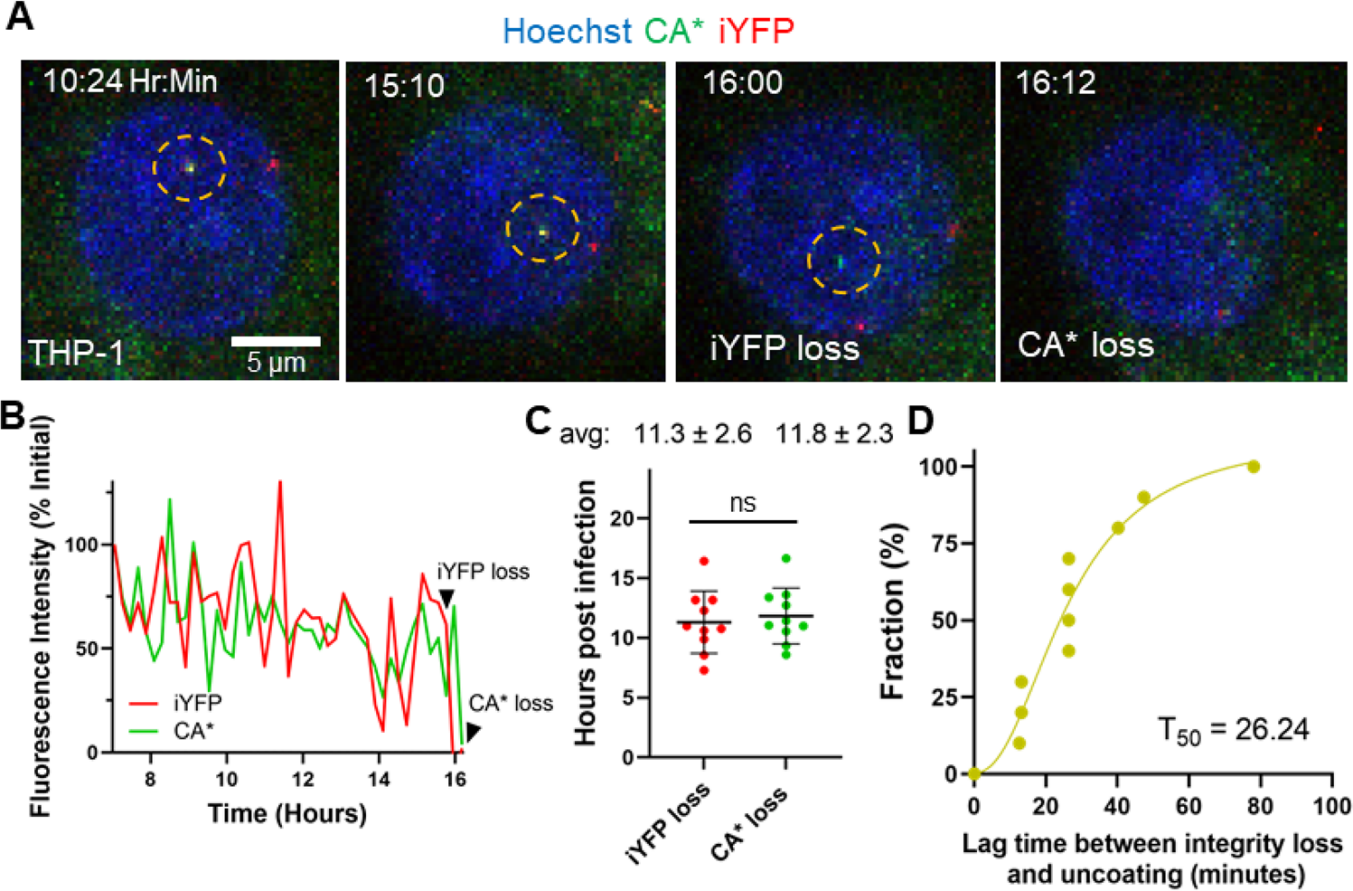
**Loss of capsid integrity precedes terminal uncoating in THP-1 derived macrophages. (A)** Representative live-cell imaging micrograph of THP-1 derived macrophage cell (stained with 2 μM Hoechst33342) containing a single CA*:Gag-iYFP core undergoing loss of integrity (iYFP) and subsequent terminal uncoating (loss of CA*). Integrity loss and uncoating listed in frame and CA*:Gag-iYFP core are highlighted by yellow dashed circle. Temporal resolution is ∼12 min per frame. **(B)** Single particle intensity trace of the CA*:Gag-iYFP core from panel A. Integrity loss and uncoating times are marked with arrows. **(C)** Integrity loss and uncoating times in THP-1 derived macrophages. Mean and SD are plotted. Average values and standard deviations are listed above each column. **(D)** Cumulative distribution plot for Δ*t* values for loss of integrity and uncoating for single particles.

To generalize the observed HIV-1 uncoating dynamics in the nucleus of transformed GHOST cells, we examined the core integrity loss and CA* disassembly in differentiated THP-1 macrophages-like cells, which model the nuclear environment of physiologically relevant cells. The HIV-1 core integrity loss and uncoating events in the nucleus of THP-1 derived cells were difficult to visualize within our imaging window due to SAMHD1 activity in these cells, which reduces the dNTP pool^26,88,89^ and slows down reverse transcription and, thereby delays uncoating.^89,90^ We were nonetheless able to detect single core uncoating events in these cells by overnight imaging, but, due to the slow rate of reverse transcription, the visualization of uncoating was likely limited to early events. Like in GHOST cells, HIV-1 cores in the nucleus of THP-1 macrophages underwent two-phase uncoating–release of iYFP followed by terminal loss of CA* (n = 10, Figure 7A, and Movie S6). The representative single particle intensity traces (Figure 7B) depict the temporal relationship between loss of capsid integrity and terminal uncoating occurring ∼12 min after release of iYFP. For particles that do uncoat during our imaging time, the average times of integrity loss is 11.3 hpi, and the subsequent terminal uncoating events are at 11.8 hpi (Figure 7C). This is consistent with the functional uncoating start time in THP-1 macrophages deduced by blocking the HIV-1 import through the nuclear pore at different times postinfection and exposing nuclear cores to a high concentrations of PF74.^36^ These results demonstrate that sequential core integrity loss and uncoating occur in the nucleus of physiologically relevant cells. Importantly, the average observed lag time between capsid integrity loss and terminal core uncoating was 31 min (Figure 7D), which is strikingly close to that in GHOST-SNAP-lamin cells (Figure 4F). Thus, the lag time for integrity loss and loss of CA* is likely independent of the cell type.

### HIV-1 Core Uncoating in the Nucleus Correlates with Productive Infection

**Figure 8:**
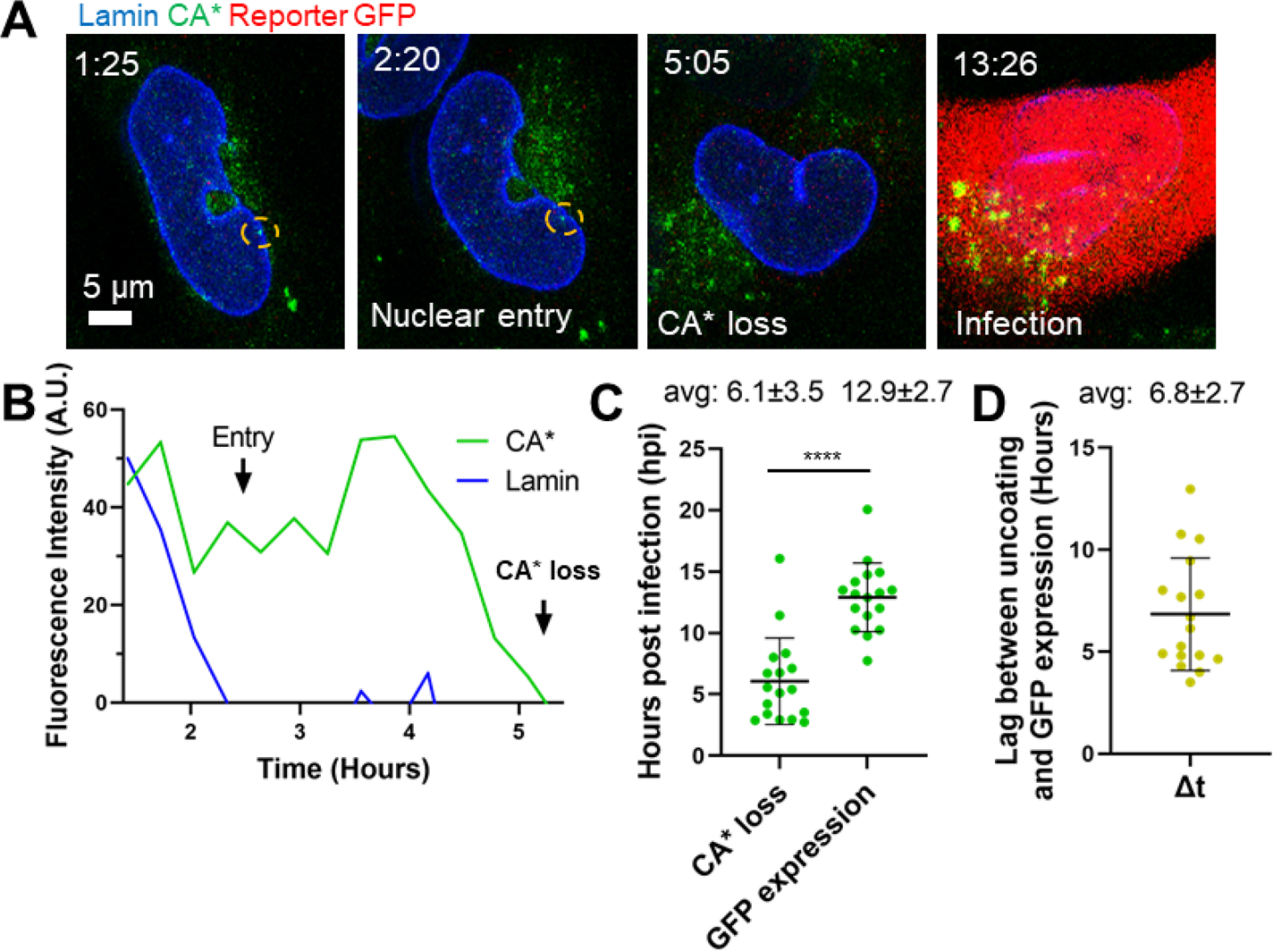
Nuclear CA* cores undergo terminal uncoating that culminates in productive infection. (A) Representative micrographs of a single CA* core entry into the nucleus, uncoating, and the resulting productive infection (GFP expression, red). The CA* core is marked by the yellow dashed circle. Temporal resolution is ∼18 min per frame. **(B)** Single particle intensity trace of the nuclear CA* core from panel A. Nuclear entry and uncoating are marked with arrows. **(C)** Distributions of functional uncoating times and times of detectable GFP reporter expression (*n* = 17). The average uncoating time and GFP reporter expression time with standard deviations are listed above the graph with SD. Statistical analysis performed with Mann–Whitney rank sum test, *p* = 0.0001. **(D)** The lag time (Δ*t*) between uncoating and GFP reporter expression in single cells. The average lag time is listed above the graph with SD.

To determine if the nuclear uncoating events correlate with productive infection, Tat-driven GFP-expression in GHOST-SNAP-lamin cells was assessed by infection with CA* GFP reporter pseudoviruses using low MOIs of 0.1–0.15. We used GFP reporter pseudovirus in combination with GFP reporter GHOST-SNAP-lamin cells to decrease the waiting time required for detection of GFP expression, since there should be more GFP produced upon Tat expression. We observed terminal loss of the CA* cores in the nucleus that correlated with productive infection through GFP reporter expression (Figure 8A, B, and Movie S7). Approximately 29% of nuclear uncoating events resulted in GFP reporter expression. This relatively low percentage of productive infection suggests that not all terminal uncoating events culminate in infection but may also be due to the limited imaging window (20 hpi) that may miss late GFP expression events. While longer live-cell imaging is possible, it presents severe practical challenges, such as phototoxicity, photobleaching, and exceedingly large file sizes. The average waiting times for infectious uncoating events was 6.1 hpi (Figure 8C), while the average time for GFP reporter expression was 12.9 hpi, with an average lag time between CA* uncoating and GFP expression being 6.8 h (Figure 8D). As expected from incomplete (∼60%) colocalization of CA* and p24 signals in coverslip-adhered pseudoviruses (Figure 1C), GFP expression was also observed in cells with no detectable nuclear CA* cores/uncoating events. Due to imperfect colocalization of CA* and p24, we cannot rule out possible infection correlations arising from unlabeled CA pseudoviruses in the prep. However, using lower MOIs, like the ones used in this study, reduces the risk of this occurring.

To assess if reverse transcription is completed prior to terminal uncoating GHOST-SNAP-lamin, we measured the kinetics of reverse transcription of CA* cores in GHOST-SNAP-lamin cells through a Nevirapine (NVP) time-of-addition assay to block reverse transcription at different time points.^25,26^ The time course of escape of CA^WT^ and CA* pseudovirus infections from NVP was very similar, demonstrating that the kinetics of reverse transcription is not affected by CA* labeling (Figure S12). The shorter *T*_50_ for virus escape from NVP compared to that for loss of CA* signal in GHOST-SNAP-lamin cells (Figure 4E) implies that completion of reverse transcription precedes terminal uncoating in the nucleus.

## Discussion

Here, we implemented a dual labeling scheme to visualize the loss of integrity and uncoating of single HIV-1 capsid in live cells, in the context of productive infection. Virions containing a ∼1.3:1 mixture of WT and amber mutant CA exhibit nearly unaltered infectivity, maturation, capsid stability, host-factor binding, and nuclear import. This direct labeling strategy, combined with incorporation of a fluid phase marker of capsid integrity (iYFP), reveals the dynamics of single HIV-1 uncoating in the cytosol, NPC, and nucleus of living cells. CA* fluorescence intensity analysis of A14-SiR-labeled CA in fixed cells revealed that cytosolic nuclear cores displayed no significant difference in CA* signals. This result supports the notion that intact or nearly intact HIV-1 cores can enter the nucleus and is fully consistent with our data (Figure S9A-C). Through live-cell imaging, we consistently observe HIV-1 uncoating proceeding through at least two distinct steps, (1) loss of iYFP, apparently through a local defect in the capsid lattice, and (2) a loss of CA* fluorescence (terminal uncoating) after a significant delay.

Our laboratory has previously developed the tetrameric CypA-DsRed (CDR) marker that incorporates into viral particles by tightly binding to HIV-1 CA at the cyclophilin A binding loop.^26,30,49^ We have observed a terminal loss of CDR at the nuclear pore roughly 20 min prior to nuclear entry.^26,30,49^ Thus, loss of core-bound CDR prior to nuclear import appears distinct from loss of iYFP or CA* in the nucleus. A possible explanation is that CDR is displaced from the cyclophilin A binding loop by Nup358, which has been reported to bind to this loop,^5,8^ or other CA-interacting nucleoporins during transport through the nuclear pore. The inner diameter of the nuclear pore is ∼60 nm,^38^ which is close to the width of capsid.^38^ However, the pore is filled with a dense meshwork of FG nucleoporins that likely pose a significant barrier for HIV-1 core import,^13^ leaving little room for additional proteins coating the capsid.

The size of an initial defect in the capsid lattice, and, therefore, the extent of CA loss upon iYFP release from the core is not known. In principle, even loss of a single hexamer (roughly 9–10 nm)^91,92^ is sufficient to release iYFP (∼4 nm). The lack of detectable decrease in CA* signal at the time of iYFP release (Figure 2I, 4H) is consistent with a relatively small defect in capsid lattice. However, given the lower signal/background ratio (3–7:1, Figure S13) in live-cell imaging settings, smaller changes to CA* (∼25%) intensity might remain undetected. We thus cannot rule out the possibility that larger defects may be responsible for iYFP release and that the vDNA is released through these defects. However, the fact that iYFP release and subsequent degradation of nuclear HIV-1 cores induced by PF74 are associated with inhibition of productive infection (Figure 1D & 5A-C)^6,27,65,81^ argues against this possibility. Although it is unclear whether PF74-induced defects are of the same size and abundance as local defects forming naturally prior to uncoating, the inhibition of infection by PF74 is inconsistent with the vDNA release from HIV-1 cores prior to terminal uncoating, which would spare vDNA from degradation and allow integration into host genome.

Several lines of evidence support the ability of the HIV-1 capsid lattice to tolerate relatively large defects without fully disassembling.^67,86,87,93,94^ Atomic force microscopy experiments suggest that HIV-1 uncoating *in vitro* is driven by reverse transcription and proceeds through a localized rupture at regions of high curvature of the capsid lattice.^67,86,87,93,94^ This conclusion is supported by course-grained and all-atom molecular dynamics simulations revealing that capsid integrity loss/breakage during reverse transcription occurs in regions of high curvature/strain.^94^ Our results support the notion that reverse transcription accelerates integrity loss and thereby increases the efficiency of uncoating, without significantly reducing the lag between integrity loss of uncoating (Figure 6A, B, C). It thus appears that vDNA synthesis may mechanically strain the capsid and promote small defect formation that may (at least partially) relieve the stress, whereas subsequent dissociation of the capsid lattice may be driven by capsid-host factor interactions. Note, however, that inhibition of reverse transcription lowered the probability of uncoating without stopping this process.^25^ This further supports a role for host factor(s) within the nuclear speckle/nucleoplasm that may regulate capsid stability.

Recent CLEM data revealed the presence of broken HIV-1 capsids in the nucleus that appear to lack the vRNP-associated density but maintain the hexagonal CA lattice.^33,38^ Zila and coauthors observed release of vDNA from nuclear HIV-1 INmScarlet-labeled fluorescent cores and correlated these foci with broken/intact cores within the nucleus using CLEM and electron tomography. These results suggest that the HIV-1 capsid does not fully disassemble in the nucleus, but rather undergoes breakage/cracking and retains a major portion of the lattice. Based on the visualization of damaged capsids, it has also been proposed that the HIV-1 capsid lattice undergoes remodeling during nuclear import.^42−44^ However, retention of the releasable iYFP marker after nuclear import is inconsistent with extensive capsid remodeling/breakage, unless this process occurs without any defect formation. Furthermore, our observation that the CA* signal is lost upon terminal uncoating ∼30 min after integrity loss is inconsistent with the capsid breaking model^33,38^ and supports the full uncoating model.

It has been shown that low levels of CA protein can associate with the vRNP complex *in vitro* and *in vivo*.^44^ Single particle tracking and biochemical evidence supports the association of untagged CA with the vRNP components (NC, IN, and RT). Our control experiments revealed that only a small fraction (∼15%) of unstable K203A CA* cores retained detectable low-level fluorescence after uncoating *in vitro* (Figure S3E, D), which may correspond to vRNP associated CA*. This very low residual CA* signal is unlikely to be detected in the nucleus, since a typical signal/background ratio in our live-cell experiments is 3–7:1 (Figure S13). Our intensity analysis with HIV-1 cores demonstrates that cores in the cytosol, at the nuclear membrane, and in the nucleus have similar CA* intensity distributions (Figure S9A-C), suggesting that the CA* signal observed in the nucleus represents intact or nearly intact cores and not the residual vRNP-associated CA* pool. We therefore interpret loss of CA* signal in the nucleus as terminal uncoating of HIV-1 capsid and not loss of the vRNP-associated CA pool.

Our results agree with the nuclear uncoating models proposed previously,^25,27^ which posits that intact HIV-1 capsids enter the nucleus, and that subsequent uncoating occurs through terminal disassembly leading to productive infection (detected based upon visualizing the sites of viral gene transcription). However, the experiments leading to this uncoating model did not employ double-labeled pseudoviruses that enabled us to dissect the dynamics of single HIV-1 uncoating in the nucleus. Instead, two separate series of experiments have been performed using eGFP-CA-labeled cores or cores single-labeled with the GagiGFP fluid phase marker.^25,27^ When using a fluid phase marker for core integrity, loss of the capsid lattice has been visualized indirectly, using fluorescently tagged CPSF6 that accumulates around intact nuclear cores.^27^ This indirect detection of capsid uncoating after loss of a fluid phase marker suggested that uncoating occurs 1–3 min after integrity loss.^27^ In contrast, our dual labeling approach that enables direct visualization of these two uncoating steps reveals a rather long (∼30 min) lag between loss of capsid integrity and terminal uncoating. The marked delay in capsid uncoating after the initial loss of capsid integrity supports the notion that the capsid lattice is being stabilized by nuclear host factors, such as CPSF6.^95^ Notably, the invariance of this lag time in osteosarcoma and macrophage cell lines suggests the involvement of potentially conserved cellular processes occurring after the initial loss of capsid integrity to trigger terminal capsid disassembly, likely dependent on host factor interactions.

The notion of multistep HIV-1 uncoating in the cytosol, NPC, and nucleus is consistent with our *in vitro* uncoating data (Figure 2F, H, I). Our results are in full agreement with the report that single HIV-1 uncoating *in vitro* progresses through “capsid opening” (release of a fluid phase marker), followed by gradual loss of CA protein and culminating in sudden loss of capsid visualized based on an indirect staining with fluorescent cyclophilin A.^48^ Unlike HIV-1 uncoating in cells, the half-life of capsids *in vitro* after virus membrane permeabilization is under 12 min, and the lag time between integrity loss and uncoating is only around 70–100 s,^48^ although this lag time has been reported to nearly double in the presence of cell lysate or IP6.^48,82^ The two-step uncoating kinetics in the cytosol are comparable to *in vitro* two-step uncoating kinetics, with *T*_50_ of 2.0 and 1.7 min, respectively, while the half-lag time for the NPC-localized core uncoating is 5.7 min (Figure 3E and Figure 2I).

We note that the current labeling strategy does not reveal the fate of viral complexes after uncoating in the cytoplasm or at the NPC. However, our previous studies using IN-labeled pseudoviruses have revealed that cytosolic uncoating resulted in efficient degradation of viral complexes by proteasomes, precluding their nuclear import.^96^ Interestingly, nuclear two-step uncoating is grossly delayed >5-fold over cytosolic and NPC uncoating (*T*_50_of ∼26 min in nucleus, 5.7 min in NPC, and 2.0 min in cytosol, Figures 4F and 3E). Such marked difference in the core stability likely reflects HIV-1 interactions with host factors in the context of intact cells at different subcellular sites.^5,8,35^ The factors influencing this delay in nuclear uncoating remains unknown, but the abundant host factor CPSF6 may play a role due to its stabilizing nature on the capsid lattice, as described in.^95^ However, it is possible that other, yet unknown nuclear factors increase capsid stability. Alternatively, the longer lag time between the two uncoating steps in the nucleus may reflect a selection for more stable cores during their passage through the NPC.

**Figure 9:**
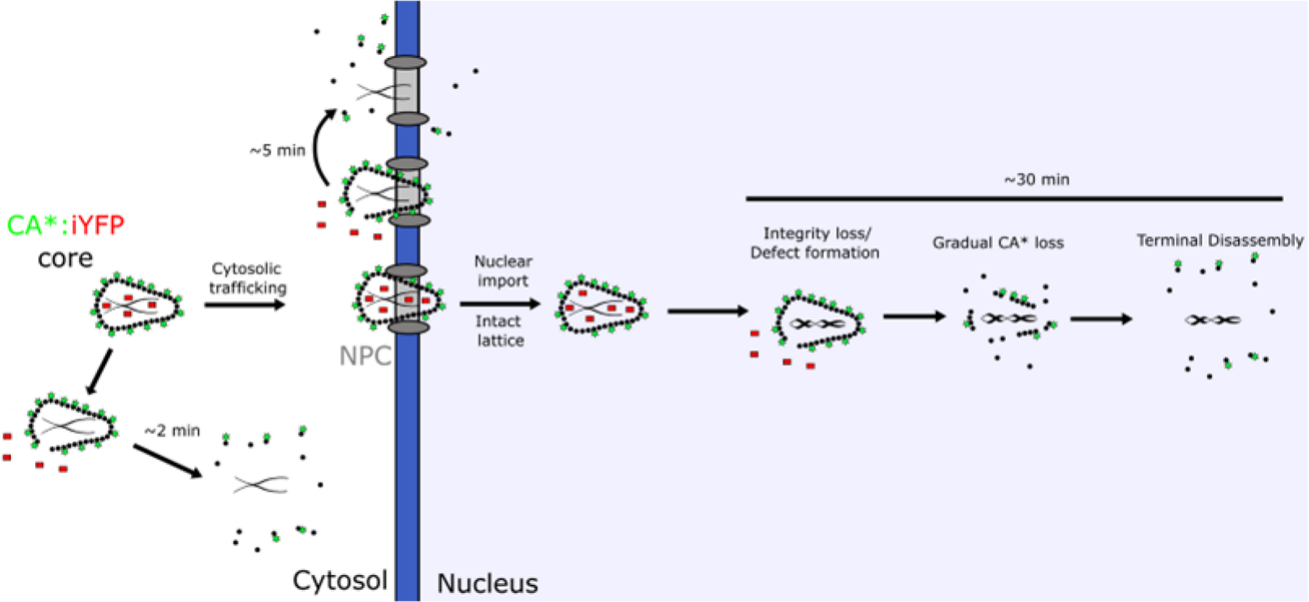
A model for HIV-1 capsid uncoating that progresses through small defect formation, gradual CA* loss, critical lattice fraction preceding terminal disassembly of the lattice in the nucleus.

The emerging model is that the intact HIV-1 capsid, which retains requisite viral enzymes for completion of reverse transcription and integration into host genome, is essential for host factor interactions that facilitate nuclear import and transport to nuclear speckles and protect the vRNA/vDNA from host-immune surveillance within the cytosol and nucleus. Premature loss of CA protein in the cytosol/nuclear membrane may expose the viral genome to innate cellular responses, including cGAS.^97^ Our study provides critical insights into the dynamic process of capsid uncoating. We hypothesize that intact capsids enter the nucleus, and uncoat via a multistep mechanism that progresses through: (1) the formation of a small defect, (2) gradual CA loss, and (3) terminal disassembly (Figure 9). Our results suggest that the HIV-1 capsid can tolerate significant loss CA, but, once the loss of CA reaches a critical point, sudden capsid disassembly occurs. These results demonstrate that HIV-1 capsid stability is tightly regulated and that small defects can trigger a sudden loss of the lattice, albeit after as significant delay. Future studies will aim to delineate the nuclear site(s) and intermediates of uncoating resulting in the release of the preintegration complex.

## Limitations of Study

The limitation of our current labeling technique lies in imperfect colocalization of CA* and p24/CA signals in the mixed pseudovirus cores. As a result, we cannot rule out the possibility that unlabeled cores uncoat outside the nucleus or that these uncoating events do not proceed through at least 2 distinct steps. However, given the minimal effects of our direct CA-labeling strategy on virus’ infectivity, interactions with host factors, and core stability, we believe that the observed uncoating of double-labeled viruses reflects productive uncoating of unlabeled HIV-1 cores. Additional limitations include a relatively low signal-to-background ratio and limited temporal resolution when imaging uncoating in live cells that preclude the detection of a small fractional CA loss and/or of any small residual CA signal after terminal uncoating. We were nonetheless able to resolve two-step uncoating events in the cytoplasm and the nucleus. Another limitation of this study is the absence of PIC/vDNA fluorescent labels in conjunction with CA*:Gag-iYFP capsids. Labeling of CA*/CA^WT^ and CA*:Gag-iYFP provides markers that are released upon uncoating, so our labeling system is blind to postuncoating events. Development of CA* pseudoviruses with labeled PIC/vDNA markers would alleviate this limitation. Further optimization of direct CA-labeling and imaging techniques should help overcome the above shortcomings.

## Conclusions

Imaging of single HIV-1 cores containing a mixture of WT CA and directly labeled CA (CA*) and colabeled with a fluid phase marker (iYFP) provides a wealth of information regarding the cellular sites, dynamics, and extent of capsid uncoating. The observation that both loss of iYFP and, after a significant delay, loss of CA* occur in the nucleus and correlate with productive infection supports the conclusion that HIV-1 uncoats in this cellular compartment and that uncoating is a multistep process. It is also found that the lags between iYFP and CA* loss are much shorter for cores in the cytosol and at NPC compared to nuclear cores. This finding demonstrates that subcellular localization of the capsid strongly modulates the uncoating process. Importantly, the lack of detectable loss of CA* at the time of iYFP release, followed by gradual decay of the CA* signal, suggests that uncoating progresses through a distinct intermediate step of small defect in the capsid lattice that triggers a cascade of events leading to terminal uncoating. The invariance of the lag time between loss of iYFP and CA* observed in divergent cell types and in the absence of reverse transcription supports the notion that processes triggered by the initial loss of capsid integrity are intrinsic to the viral core or determined by core interactions with ubiquitous nuclear factors.

## Materials and Methods

### Plasmids

The CA* amber TAG stop codon mutation was introduced into the pNL4–3 R-E- eGFP and pR9ΔEnv plasmid by amplification of the MA-CA region between the BssHII and SpeI restriction sites (forward primer: 5′ – TGCAGCaagcttGCGCGCACGGCAAGAGG – 3′, reverse primer: 5′ – GAGTCGggatccACTAGTAGTTCCTGCTATGTCACTTCCCCTTGG – 3′) and ligating the domain into the pUC19 cloning vector (Addgene plasmid # 50005), using the T4 ligase ligation kit (New England Biolabs, Cat# M020S0). Quikchange mutagenesis primers were constructed to mutate the isoleucine-91 site to an amber stop codon (TAG, forward primer for mutation: 5′-cagtgcatgcagggccttaggcaccaggccagatg-3′, reverse primer for mutation: 5′-catctggcctggtgcctaaggccctgcatgcactg-3′). Quikchange mutagenesis for A14 were (forward: 5′-tccaggggcaaatggtacatcagtagatatcacctagaactttaaatgc-3, reverse: 5′-gcatttaaagttctaggtgatatctactgatgtaccatttgcccctgga-3′), for P85 were (forward: 5′-cagaatgggatagattgcattaggtgcatgcagggcctattgc-3′, reverse: 5′-gcaataggccctgcatgcacctaatgcaatctatcccattctg-3′), and G94 (forward: 5′-gcagggcctattgcaccatagcagatgagagaaccaagg-3′, reverse: 5′-ccttggttctctcatctgctatggtgcaataggccctgc-3′).The mutated partial MA-CA domain was reinserted into the BssHII and SpeI sites of pNL4–3 R-E- eGFP or pR9ΔEnv to construct the pNL4–3ΔEnv R-E- eGFP or pR9ΔEnv CA* plasmids, respectively. WT backbone vectors were derived using the pNL4–3 R-E- eGFP construct, as described in.^6,26,30^ The pR9ΔEnv K203A CA* plasmid was constructed by digesting the pR9ΔEnv CA* plasmid with the BssHI and SpeI restriction enzymes and ligating this MA-CA fragment into the pR9ΔEnv K203A plasmid (A kind gift from Dr. Christopher Aiken). G89V CA was derived from the pR9ΔEnv G89V HIV-1 backbone vector (a kind gift from C. Aiken).^98^ The Gag-iYFP pNL4–3ΔEnv was derived from the Gag-iGFP vector,^47^ developed by Naoyuki Kondo (Kansai Medical). The pMD2.G VSV-G vector encoding for VSV-G envelope glycoprotein was obtained from John Naughton at the Salk Institute. The peRF1 E55D and PylRS NES AF amber codon suppression plasmids were a kind gift from Edward Lemke (Johannes Gutenberg University).^55^

### Cell Lines and Reagents

HEK293*T*/17 cells (ATCC, Manassas, VA), HeLa-derived TZM-bl cells ectopically expressing CD4 and CCR5^99^ (NIH AIDS Reference and Reagent Program, ARP-8129, contributed by J Kappes, X Wu), TZM-bl cells expressing TRIMCyp^100^ (kind gift from Dr. Jeremy Luban, University of Massachusetts), GHOST(3) CXCR4/CCR5 cells (NIH AIDS Reference and Reagent Program, contributed by V. KewalRamani & D. Littman) were grown in a complete high-glucose Dulbecco’s Modified Eagle Medium (DMEM, Mediatech, Manassas, VA) supplemented with 10% heat-inactivated Fetal Bovine Serum (FBS, Sigma, St. Louis, MO) and 100 U/mL penicillin-streptomycin (Gemini Bio-Products, Sacramento, CA). THP-1 monocytic cells (ATCC, Manassas, VA) were grown in complete Roswell Park Memorial Institute growth media (RMPI, Gibco) supplemented with 10% heat inactivated FBS and 100 U/mL penicillin/streptomycin. Growth medium for HEK293*T*/17 cells was additionally supplemented with 0.5 μg/mL Neomycin (Mediatech).

GHOST-SNAP-lamin cells were obtained by transduction with VSV-G pseudotyped viruses encoding for SNAP-Lamin B1 transgene. Transduction efficiency was assessed by visual inspection of SNAP-Lamin expression using the SNAP-Cell TMR-Star (NEB, #S1905S). SNAP-TMR-Star staining was performed as manufacturer’s protocol.

The noncanonical amino acid transcyclooctene lysine (TCO*A, Cat# SC-8008) was purchased from SiChem (Bremen, Germany) and dissolved at 100 mM in 15% DMSO, 0.2 M NaOH, and stored at −20 °C. The clickable Silicon Rhodamine-Tetrazine (SiR-tetrazine, Cat#SC008) dye was purchased from Spirochrome (Thurgau, Switzerland), dissolved in methanol at 1 mM, aliquoted in 20 μL evaporating with argon gas, and stored at −20 °C. Aliquots of SiR-tetrazine were redissolved in 20 μL of DMSO at a final concentration of 1 mM prior to use. The mCLING-Atto488 (Cat# 710 006AT3) membrane marker was purchased from Synaptic Systems (Gottingen, Germany), dissolved in deionized water at 50 nmol/mL and stored at −80 °C. The Bright-Glo luciferase assay kit was purchased from Promega (Madison, WI). Primary IgG antibodies specific to Lamin B1 (#ab16048) and the secondary IgG donkey antirabbit AF405 conjugated antibody (#ab175651) were purchased from Abcam (San Francisco, A). The SNAP-Cell TMR-Star and Oregon Green were purchased from New England Biolabs (NEB, #S1905S, #S9104S). Hoechst33442 was purchased from ThermoFisher (Cat# 62249). HEPES 1 M solution (Cat# SH30237.01) was purchased from Cytiva Life Sciences (Marlborough, MA). Phosphate buffered saline containing Mg^2+^/Ca^2+^ (dPBS+/+) and Mg/Ca-free (dPBS–/−) were purchased from Corning (Mediatech, Manassas, VA). The mouse anti-GFP A.V. living colors antibody was purchase from Clontech (Cat# 632381). The Alexa Fluor 568 goat antimouse IgG H+L antibody was purchased from ThermoFisher (Cat# A11004). The capsid-binding inhibitor PF74 was purchased from Sigma-Aldrich (CAT# SML0835). The HIV-1 protease inhibitor Saquinavir (ARP-4658) and the integrase inhibitor Raltegravir (HRP-11680) were obtained through the NIH HIV Reagent program, (contributed by DAIDS/NIAID). The mouse IgG anti-p24/CA specific antibody AG3.0 (donated by Dr. J. Alan),^101^ reverse transcriptase inhibitor Nevirapine (ARP-4666), integrase inhibitor raltegravir (HRP-11680), protease inhibitor (ARP-4658), Anti-HIV-1 antibody specific to p24/CA hybridoma183 (ARP#1513) and Human HIV-IgG serum (ARP#3957) were all received from the NIH HIV reagent program.

### Pseudovirus Production and Characterization

To produce CA*:Gag-iYFP dual-labeled viruses, the pNL4–3ΔEnv R-E- CA* eGFP or pR9ΔEnv I91* backbone (1.2 μg), pNL4–3ΔEnv Gag-iYFP (0.8 μg), pMD2.G VSV-G (0.2 μg), PylRS NES AF (0.5 μg), and peRF1 E55D (0.5 μg) were transfected into single wells (3.5 cm) of the Corning Costar 6-well cell culture plate containing HEK293*T*/17 cells using the JetPrime Transfection reagent (VWR, Randor, PA) according to the manufacturer’s protocol. Virions labeled only with CA* were produced using pNL4–3ΔEnv R-E- CA* eGFP/pR9ΔEnv I91* backbone (1.2 μg), pNL4–3 R-E- CA^WT^ eGFP/pR9ΔEnv CA^WT^ (0.8 μg) and the pMD2.G VSV-G, PylRS NES AF, and peRF1 E55D vectors at the same ratio as for the CA*:Gag-iYFP pseudovirus production. For TRIMCyp restriction assays, control pseudoviruses were produced using pR9ΔEnv G89V (2.0 μg) with the pMD2.G VSV-G, PylRS NES AF, and peRF1 E55D vectors at the same ratio as for the CA*:Gag-iYFP pseudovirus production. K203A CA* pseudoviruses were produced using pR9ΔEnv K203A CA* (1.2 μg), and pR9ΔEnv K203A (0.8 μg) with the pMD2.G VSV-G, PylRS NES AF, and peRF1 E55D vectors at the same ratio as described. Complete DMEM transfection medium was supplemented with 250 μM TCO*A, prediluted 1:5 in 200 mM HEPES.

To produce CA* pseudoviruses containing the A14, P85, and G94 amber codons, the CA* pR9ΔEnv vector (1.2 μg), the WT CA pR9ΔEnv vector (0.8 μg), in addition to the VSV-G and amber suppression machinery utilized in the above pseudovirus recipes. To conduct the plasmid titration assay, the CA* (A14, I91, P85, and G94) and WT pR9ΔEnv vectors were mixed at ratios of 1:0, 1:1, 1:2, 1:4, and 0:1 CA^WT^/CA*, with the VSV-G and amber suppression machinery plasmids being kept consistent.

For production of transducing viruses for SNAP-Lamin B1, the pLENTI-SNAP-Lamin B1 HIV-1 transfer vector (2 μg), psPAX2 (Addgene #12260, Kind gift from Didier Trono, 1 μg), and pMD2.G VSV-G (0.2 μg) was transfected into HEK293*T*/17 cells using the JetPrime transfection kit as per manufacturer’s recommendation.

After 16 h of transfection, the growth medium was replaced with 2 mL of complete DMEM without phenol red, supplemented with 250 μM TCO*A. After the media change, the cells were incubated for an additional 32 h at 37 °C in 5% CO_2_. After a total of 48 h, the pseudovirus-containing supernatant was collected, filtered through a 0.45 μm filter, concentrated 10x using the Lenti-X lentivirus concentrator (Clontech Laboratories, Inc. Mountainview, CA), resuspended in dPBS+/+, and stored at −80 °C. Pseudovirus p24 content was quantified from viral preparations using the p24 ELISA assay.^102^ The multiplicities of infection (MOIs) were determined in GHOST-SNAP-lamin cells by examining the percent of GFP positive cells after 48 h postinfection with VSV-G CA* pseudoviruses using a BioTek Cytation 3 imaging plate reader (instrument use is courtesy of Dr. Baek Kim).

### Pseudovirus SPIEDAC Click-Labeling

TCO*A-labeled CA*:Gag-iYFP and CA* pseudovirus cores were labeled in cells post/during virus-cell fusion. Viral cores were labeled at 0, 30, and 60 min postinfection for GHOST-SNAP-lamin and THP-1 cells, respectively, by incubating cells with 250 nM SiR-tetrazine in FluoroBrite DMEM (Gibco, CAT#A1896701) without FBS for 20 min. Cells were washed once with dPBS+/+, and further incubated in complete FluoroBrite medium.

### Single-Round Infectivity and Restriction Assay

Virus infectivity was measured by luciferase reporter activity in TZM-bl cells or TZM-bl cells expressing exogenous TRIMCyp. TZM-bl cells were plated in glass-bottom 96-well plates and infected 12–20 h after plating with VSV-G pseudotyped viruses, typically, at 1:100 and 1:1,000 dilutions of the initial stock. Virus-cell binding was facilitated by centrifuging cells with virus-containing solutions for 30 min at 1550xg and 4 °C. Pseudovirus SiR-tetrazine click labeling was performed 30 min post infection, as described above. At 48 hpi, cells were lysed, and the luciferase activity was quantified using the Bright-Glo luciferase substrate (Promega) using the supplier’s protocol. Raw luciferase values were normalized to pseudoviral p24/CA content to derive specific infectivity. GHOST-SNAP-lamin cells were plated and infected with VSV-G pseudotyped HIV-1 CA* and CA^WT^ virions. Pseudoviruses were centrifuged onto cells by the same method described in the luciferase protocol. After 30 min postinfection, pseudovirus containing cells were labeled with SiR-tetrazine as described above and incubated in complete DMEM for an additional 47 h. At 48 hpi, cells were stained with 2 μM Hoechst33442 for 10 min, washed with dPBS, then fixed with 4% PFA (Electron Microscopy Sciences, Cat# 1570-S) in dPBS for 20 min. Nine adjacent fields of view were acquired for each well, and the number of GFP positive cells and cell nuclei, stained with Hoechst33442, were quantified using the cell segmentation protocol within the BioTek plate reader software.

### Western Blotting

HIV-1 CA* and CA^WT^ pseudoviruses suspension (20 pg p24) was heated to 95 °C, reduced with ß-mercaptoethanol, then loaded ono a 4–15% precast SDS-PAGE gel, with the Precision Plus Protein kaleidoscope ladder (BioRad, Cat# 1610375). The contents of the SDS-PAGE gel were transferred to a 0.45 NC nitrocellulose membrane (Cytiva, Cat# 10600012) and blocked with 10% skimmed milk in PBST (PBS+/+ with 0.1% tween-20). The membrane was then incubated with the HIV-IgG human serum antibodies (1:2,000) and/or the mouse monoclonal anti-GFP living colors IgG antibody (1:1,000) for 1 h at room temperature or overnight at 4 °C. After three 5 min washes with PBST, the membrane was incubated with the secondary IgG donkey antimouse IRDye 800CW conjugated antibody (1:10,000) (Li-Cor, Cat# 926–32212) or goat antihuman IRDye 800CW (1:10,000) (Li-Cor, Cat# 926–32232). The membrane washes were repeated, and the membrane was maintained in deionized water. The membrane was visualized using a Li-Cor Oddessy Clx fluorescent gel imager, using the 700, and 800 emission detectors at a resolution of 169 μm for band visualization.

For CA*:WT and CA*:Gag-iYFP stoichiometry determination, 50 μg of lysates of HEK293*T*/17 cells transfected with 1.2 μg CA* or 0.8 μg CA^WT^/iYFP backbone (with proper amber suppression machinery) in the presence of 200 nM Saquinavir were immunoblotted, as described above. Protein concentrations in cell lysates were quantified using the Micro BCA protein assay kit (ThermoFisher, Cat# 23235). On a separate nitrocellulose membrane, GAPDH was immunoblotted with the Rabbit anti-GAPDH polyclonal antibody (1:1000, ThermoFisher, Cat# PA1–987), and visualized with the IRDye 800CW Donkey anti-Rabbit IgG secondary antibody (Licor, Cat# 9926–32213).

### THP-1 Macrophage Differentiation

For visualization of CA*:Gag-iYFP uncoating in THP-1 derived macrophage cells, 100,000 THP-1 cells were treated with 125 nM phorbol 12-mysristate 13-acetate (PMA, Sigma-Aldrich, CAT# P1585) in complete RPMI-1640 medium for 24 h. After 24 h, PMA containing medium was removed, and cells were incubated in complete RMPI-1640 medium for an additional 24 h.

### Single Virus Imaging *in Vitro*

Unlabeled CA* and CA^WT^ pseudoviruses were centrifuged onto poly-l-lysine treated #1.5 8-well chambered cover glass slides (ThermoScientific, Cat# 155409) for 15 min at 1550xg, 4 °C. The chambers were washed 1x with dPBS+/+, blocked with 20% FBS in dPBS+/+ for 30 min at room temperature. After blocking, 250 nM SiR-tet was added to the immobilized pseudoviruses in 10% FBS in dPBS+/+ for 20 min at 37 °C, followed by an additional washing step with DPBS+/+. The fluorescently labeled pseudoviruses were imaged on a Zeiss LSM880 confocal microscope using a C-Apo 40*x*/1.3 NA or 63*x*/1.4 NA oil objective or on a GE Healthcare DeltaVision widefield epifluorescence microscope. Multiple fields of view were acquired using the highly attenuated 488, 561, 633 nm laser lines, with respective EYFP, TMR, and Alexa Fluor 647 emissions bands collected using the Gallium-Arsenide (GaAsP) spectral detector for the LSM880. For *in vitro* uncoating assays, viral membranes were permeabilized with 100 μg/mL saponin (Riedel de-Hähn) after 5–10 image frames acquired every 5 s or after 1 frame being imaged every 30 s, and viruses were imaged for 25–30 min in the presence of permeabilizing agents.

### Immunofluorescence and Fixed Cell Imaging

For fixed-cell imaging, GHOST-SNAP-lamin cells were plated onto collagen or poly-l-lysine treated #1.5 8-well chamber slides and infected with CA* or CA^WT^ at MOI of 0.2–0.5 via centrifugation. At 30 min post infection, cells were labeled with 250 nM SiR-tetrazine in FluoroBrite DMEM without FBS then washed with dPBS+/+, as described. GHOST-SNAP-lamin cells were labeled with SNAP-TMR-Star or SNAP-Oregon Green for 20 min before infection, using the supplier’s protocol. Cells were incubated at varying times post infection and fixed with 4% PFA for 20 min at room temperature. PFA was quenched with 20 mM Tris in dPBS+/+, and cells were either imaged immediately in dPBS+/+ or subjected to immunofluorescence labeling. For the latter protocol, cells were permeabilized with 0.25% Triton X-100 (Sigma-Aldrich, Cat# X100–100 mL) for 5 min at room temperature, washed three times with PBST, and blocked with 20% FBS in PBST. After blocking, rabbit anti-LaminB1 antibodies (1:1,000, Abcam #ab16048) in blocking buffer were added to the cells for 1 h at room temperature, washed three times with PBST, followed by the addition of the antirabbit AF405 secondary antibody (1:1,000, Abcam #ab175651) for 1 h at room temperature. After secondary antibody incubation, the cells were washed with PBST three times and imaged in dPBS+/+.

For experiments using mCLING-Atto488, GHOST-SNAP-lamin cells were labeled with 2 μM mCLING-Atto488 for 5 min prior centrifuging with pseudoviruses in the presence or mCLING-Atto488, as described above. Virus-decorated cells were washed with DMEM to remove unbound viruses and excess mCLING-Atto488 and incubated in DMEM until labeling with SiR-tetrazine, as described above.

### Live-Cell Imaging of HIV-1 Infection

Single HIV-1 capsid integrity loss, uncoating, and subsequent infection were visualized by live-cell imaging using confocal microscopy. 50,000 GHOST-SNAP-lamin cells were plated onto collagen coated 35 mm #1.5 cover glass imaging dishes (Matek, CAT# P35G-1.5–10-C) and infected at varying MOIs (0.1–0.15 for correlating uncoating with infection, 0.2–0.5 for visualization of uncoating for a shorter time, without correlating with infection) with VSV-G pseudotyped CA* NL4–3 eGFP particles copackaged with either pNL4–3 CA^WT^ eGFP/psPAX2 or NL4–3 GagiYFP. Virus binding to cells was enhanced via centrifugation (1550xg, 30 min, 4 °C). Prior to infection, GHOST-SNAP-lamin cells were labeled with SNAP-Cell TMR-Star or Oregon Green, as per supplier’s protocol. After centrifugation, cells were labeled with SiR-tetrazine, as described. Cells were then washed with dPBS+/+ and incubated in FluoroBrite DMEM with 10% FBS and 20 mM HEPES in a Zeiss LSM880 microscope equipped with temperature-, humidity- and CO_2_-controlled environmental chamber. Cells were maintained in 10 μM aphidicolin (Sigma-Aldrich, CAT# A0781–5 mg) to block cell division. 3D time-lapse imaging was performed with a Zeiss LSM880 laser scanning confocal microscope using a C-Apo 40*x*/1.3NA oil immersion objective. All live-cell imaging experiments were conducted using the highly attenuated 488, 514, 561, and 633 nm laser lines with the Gallium-Arsenide (GaAsP) spectral detectors with 0.21 or 0.23 μm pixel size and the pinhole size of ∼4.0 Airy Units (AU).

To track nuclear import of CA* cores, cells were imaged at 1 h postinfection, for a total of 2–3 h every ∼1–2 min using a tile scan of 16 adjacent fields-of-view with 5–7 Z-stacks spaced by 0.7 μm. To track relationship between uncoating and productive infection, live-cell imaging was performed from 1.5 to 2 hpi up to 24 h postinfection, with 100 adjacent fields-of-view acquired using 9–11 Z-stacks spaced by 0.5 μm every ∼18 min. Axial drift was compensated using the Carl Zeiss DefiniteFocus module. To track integrity loss and uncoating, 49 adjacent fields-of-view were imaged every 5–10 min for a total of 20 h postinfection with 9–11 Z-stacks spaced by 0.5 μm.

To track two-step cytosolic uncoating of CA*:Gag-iYFP cores, cells were infected with ∼300 pg CA*:Gag-iYFP pseudoviruses, as described previously. CA*:Gag-iYFP infected cells were labeled with 250 nM SiR-tetrazine immediately after virus binding for 20 min at 37 °C with 5% CO_2_ for 20 min. Cells were imaged at 0.5 hpi for 1 h increments with ∼10 s temporal resolution with 10 Z-stacks spaced 0.5 μm. Imaging was carried out with a C-Apo 63*x*/1.4NA oil immersion objective.

To track the integrity loss and uncoating events in THP-1 derived macrophages, 100,000 THP-1 cells were differentiated with 125 nM PMA on Poly-l-Lysine, as described above. THP-1 macrophages stained with 2 μM Hoechst33342 were infected with 200 pg CA*:Gag-iYFP pseudoviruses and labeled with SiR-tetrazine, as described above. Live-cell imaging experiments began at ∼1.5 or ∼7 hpi. Sixty-four adjacent fields-of-view were acquired every ∼12 min.

### Image Analysis

To analyze colabeling efficiency and *in vitro* uncoating kinetics of CA*, iYFP-labeled and p24-immunotsained HIV-1 cores, three random fields of view were processed with the ComDet c.0.5.5 ImageJ plugin. Single pseudovirus particles were detected with 3-pixel size spot detection and 3–5 intensity thresholding. The *in vitro* uncoating kinetics was plotted as the number of CA* and iYFP-labeled particles over time normalize to the initial number of double-positive particles. Immature pseudoviruses and stable cores that did lose integrity or undergo uncoating were excluded from analysis.

To analyze mCLING-Atto488-labeled GHOST-SNAP-lamin cells for CA* fluorescence intensity distributions, the ICY bioanalysis (http://icy.bioimageanalysis.org) protocol development tool was used. Each individual channel (CA*, Lamin, mCLING-Atto488) was used for segmentation. mCLING-Atto488 colocalized CA* cores were eliminated with the subtract ROI tool by segmenting mCLING-Atto488 foci and removing them from the image. Nuclear membrane-localized cores were detected by HK-means detection in ICY with a binary mask overlaid onto the Lamin signal and used as the detection mask for the spot detector plugin. Intranuclear cores were detected by convexifying Lamin ROIs and calculating the space within the Lamin fluorescent signal using the erode ROI processor. Cytosolic cores were detected by subtracting nuclear and nuclear membrane fluorescence from the images and using the spot detector plugin tool to detect all non-nuclear and mCLING-Atto488(−) cores. All CA* ROIs had an additional 2 pixels dilated from their detection foci to quantify and subtract the local background fluorescence intensity for each particle.

Single particle tracking and live-cell imaging experiments were analyzed using the Zeiss Zen Black software to visually determine the time of intranuclear uncoating and integrity loss events. Live-cell micrographs were annotated to list the uncoating/integrity loss times with the text editor tool within the Zen Black software. To track the single particle fluorescence intensity over time, the ICY spot tracking/manual tracking plugin was used due to the high mobility and nuclear rotation of GHOST-SNAP-lamin cells. Micrographs were first reduced to 2D by cropping cells of interest, extracting Z-planes containing particles of interest, and using maximum intensity projections for single particle tracking using ICY. The ICY track manager intensity profile and background subtraction processer was used to make intensity traces. Intensity traces were normalized to the initial fluorescence intensity value, when applicable. Ensemble intensity trace analysis was conducted by normalizing fluorescent CA* values to the time of iYFP loss *in vitro* and within the nucleus of cells.

GHOST-SNAP-lamin cells have high degrees of mobility. To correct this motion in live-cell imaging experiments, the TrackMate V7 ImageJ tool was used. Cells of interest were imported into the TrackMateV7 tool, and individual Hoechst33342 and Lamin fluorescent nuclei were detected using the LoG detector with 15–30 μm object segmentation. Individual segmented nuclei were tracked using the simple LAP tracker tool with varying gap-closing parameters for each cell. Individual cell tracks were selected, and the track stack processing tool was used to superimpose each time frame to center the cell-of-interest in the middle of the movie’s field-of-view. The subsequent movies were then processed through ICY or Zeiss Zen Black software.

### Statistical Analysis

Statistical analysis was carried out using GraphPad Prism (9.3.1) or MatLab (R2021a). All infectivity-based data were analyzed using the Student’s unpaired *t* test of Prism. Fluorescence intensity data with non-normal distributions were analyzed with Prism using the Mann–Whitney rank sum nonparametric test. For the mCLING-Atto488 intensity distribution experiments, custom MatLab scripts were used to analyze data with a nonparametric Kolmogorov–Smirnov test with optimal binning to account for large differences in population sizes. Uncoating curves were quantified in Prism using the nonlinear curve fitting processor with the one-phase decay equation (*Y* = (*Y*0 – *Plateau*) * exp (− K*X) + *Plateau*. PF74 time-of-addition experiments were curve-fitted using the nonlinear regression curve fitting processor with the Sigmoidal equation (*Y* = *Bottom* + (*X* ^ *Hillslope*) * (*Top* – *Bottom*)/(*X*^*HillSlope*^ + *EC*50^*HillSlope*^). Cytosolic and *in vitro* uncoating lag times were curve fitted with the one-phase association equation *Y* = *Yo* + (*Plateau* – *Yo*) * (1–exp (K*x)). Linear slope fitting for ensemble intensity analysis was performed with the *Y* = *mx* + *b*. Statistical significance was assigned as *p* < 0.05 (*), *p* < 0.01 (**), *p* < 0.001 (***), *p* < 0.0001 (****) respectively. *T*_50_ values for nuclear uncoating lag time distributions were curve-fitted with the sigmoidal equation Y = 100*(X ^ HillSlope)/(EC50 ^ HillSlope + (X ^ HillSlope)).

## Supporting Information Available

Additional validation experiments for CA* pseudoviruses, additional control experiments/analyses, and single particle intensity traces (Figures S1–S13) (PDF)
Movie S1. Cytosolic uncoating progresses through two steps (AVI)
Movie S2. Nuclear periphery-localized uncoating progresses through two steps (AVI)
Movie S3. Live-cell imaging of CA* nuclear import (AVI)
Movie S4. Multistep nuclear HIV-1 uncoating in a GHOST-SNAP-Lamin cell (AVI)
Movie S5. PF74 leads to release of core-trapped fluid phase marker and results in capsid degradation (AVI)
Movie S6. Multistep uncoating in the nucleus of THP-1 macrophage-like cells (AVI)
Movie S7. Nuclear uncoating correlates with productive infection (AVI)

## Supplementary Material

nn3c07678_si_001.pdf

nn3c07678_si_002.avi

nn3c07678_si_003.avi

nn3c07678_si_004.avi

nn3c07678_si_005.avi

nn3c07678_si_006.avi

nn3c07678_si_007.avi

nn3c07678_si_008.avi

## Notes

The manuscript was previously uploaded onto the BioRxiv preprint server. Levi B. Gifford and Gregory B. Melikyan. “Human Immunodeficiency Virus 1 Capsid Uncoating in the Nucleus Progresses Through Defect Formation in the Capsid Lattice”. BioRxiv. 2023. 2023.08.22.553958. doi: 10.1101/2023.08.22.553958 (accessed 01/12/2024).
