# Supplementary material for: HIV-1 Capsid Uncoating Is a Multistep Process That Proceeds through Defect Formation Followed by Disassembly of the Capsid Lattice
Source: ACS Nano. 2024 Jan 19;18(4):2928–47. doi: 10.1021/acsnano.3c07678 (PMC10832047; doi:10.1021/acsnano.3c07678)
Supplement: Supplementary file 1 — nn3c07678_si_001.pdf [file nn3c07678_si_001.pdf]

HIV-1 capsid uncoating is a multistep process that proceeds through defect formation followed by disassembly of the capsid lattice

Levi B. Gifford[1], and Gregory B. Melikyan[1,2*]

[1]Department of Pediatrics, Emory University School of Medicine, Atlanta, GA 30322, USA
[2]Children's Healthcare of Atlanta, Atlanta, GA 30322, USA.

Supplementary Figures, Movies and Legends

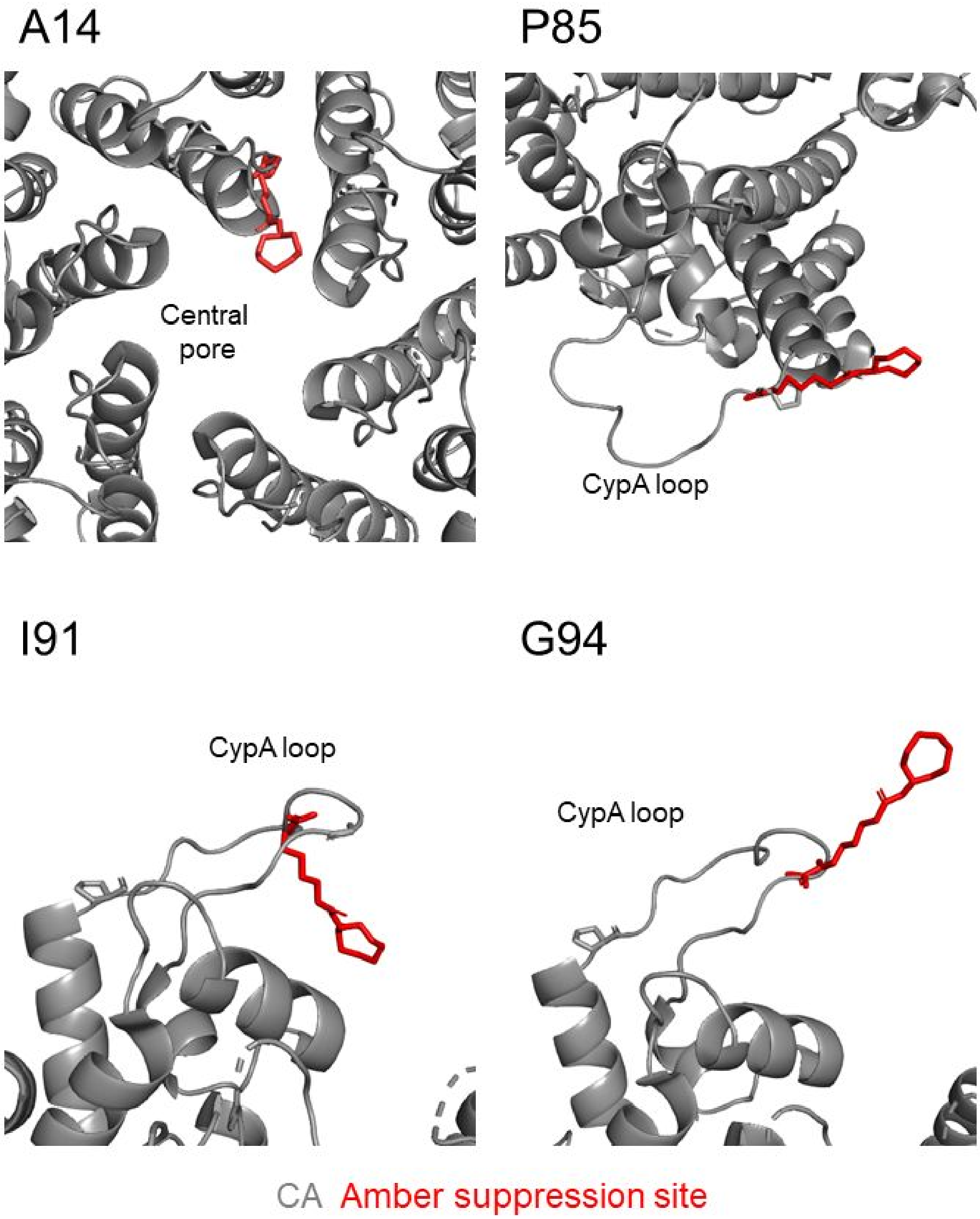

Fig. S1. CA amino acid positions for amber codon suppression. Structurally predicted models for TCO incorporation into CA hexamers using the 3MGE and 5KAX PDB models. Modeling was conducted by superimposing the TCO group at the A14, P85, I91, and G94 positions by matching the orientation and steric positions of the amino acid backbone groups. The inserted 5FAX TCO group is colored red and the 3MGE CA hexamer is colored grey. Central pore and cyclophilin binding loop regions are annotated with black text.

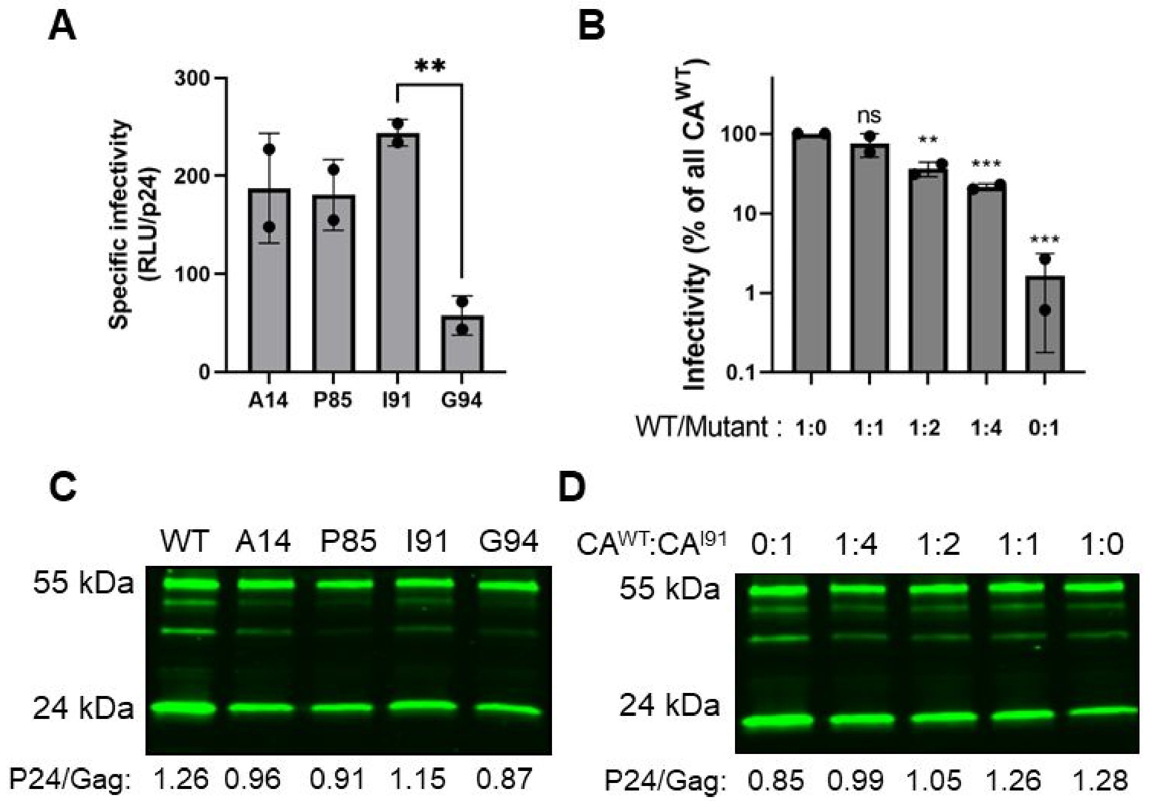

Fig. S2. Selection of I91 amber suppression site and pseudoviral production ratio. A) comparison of specific infectivity of all four amber suppression sites, all complemented with CA^{WT} at a 1.5:1 CA^{TAG}:CA^{WT}. Mean and SD plotted for two independent viral preparations. **B)** CA^{I91} and CA^{WT} plasmid titration experiment to determine the optimal plasmid ratio for pseudovirus production. Specific infectivity was obtained by normalizing the luciferase signal to viral p24 content and plotted as % of the 1:0 CA^{WT}:CA^{I91} control. Mean and SD plotted for two independent viral preparations. **C)** Pseudovirus maturation efficiency for CA^{WT}, A14, P85, I91, and G94 CA* pseudoviruses (20 pg of pseudovirus was used, as described above). **D)** I91 plasmid titration to derive proper plasmid ratio for optimal pseudoviral production recipes. I91 CA* and CA^{WT} plasmids were titrated at ratios of 0:1, 1:1, 2:1, 4:1, and 1:0 CA*:CA^{WT} to determine a useable plasmid ratio for virus production. Western blot detection was carried out using the same detection methods described above.

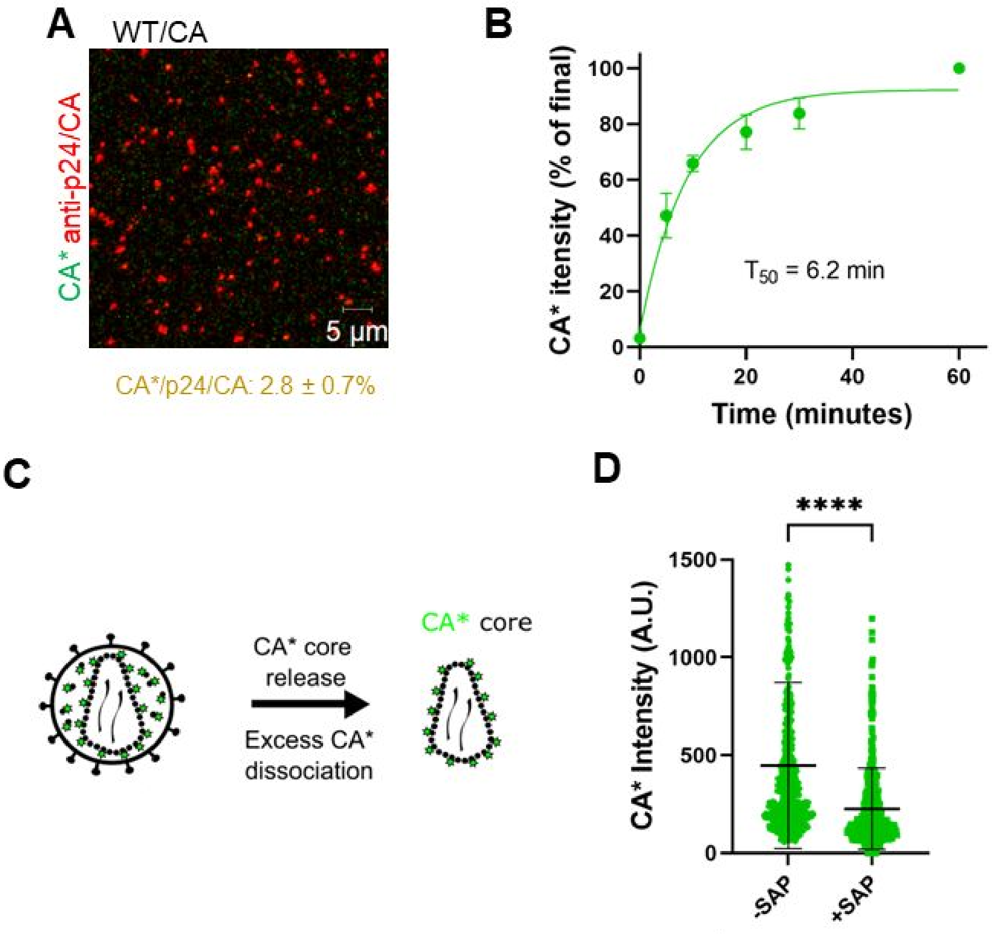

Fig. S3. Additional validation of CA* pseudoviruses. **A)** Cell-free WT pseudovirus plated on poly-L-lysine coated coverslip, labeled with 250 nM SiR-tetrazine and immunostained with mouse anti-p24 AG3.0 antibody and anti-mouse AF568 second antibody. Mean and SD are listed. **B)** Kinetics curve for tetrazine labeling of CA* pseudoviruses. Cell-free CA* pseudoviruses were stained with SiR-tetrazine for 0, 5, 10, 20, 30, and 60 minutes to determine the optimal time for tetrazine labeling. Single exponential curve fit used with means and SD from two independent experiments. **C)** Representative illustration demonstrating the loss of non-lattice incorporated CA* upon fusion/lysis. **D)** Fluorescence intensity of cell-free viruses labeled with SiR-tetrazine and permeabilized with 100 µg/mL Saponin ($p < 0.0001$, Mann-Whitney rank sum test).

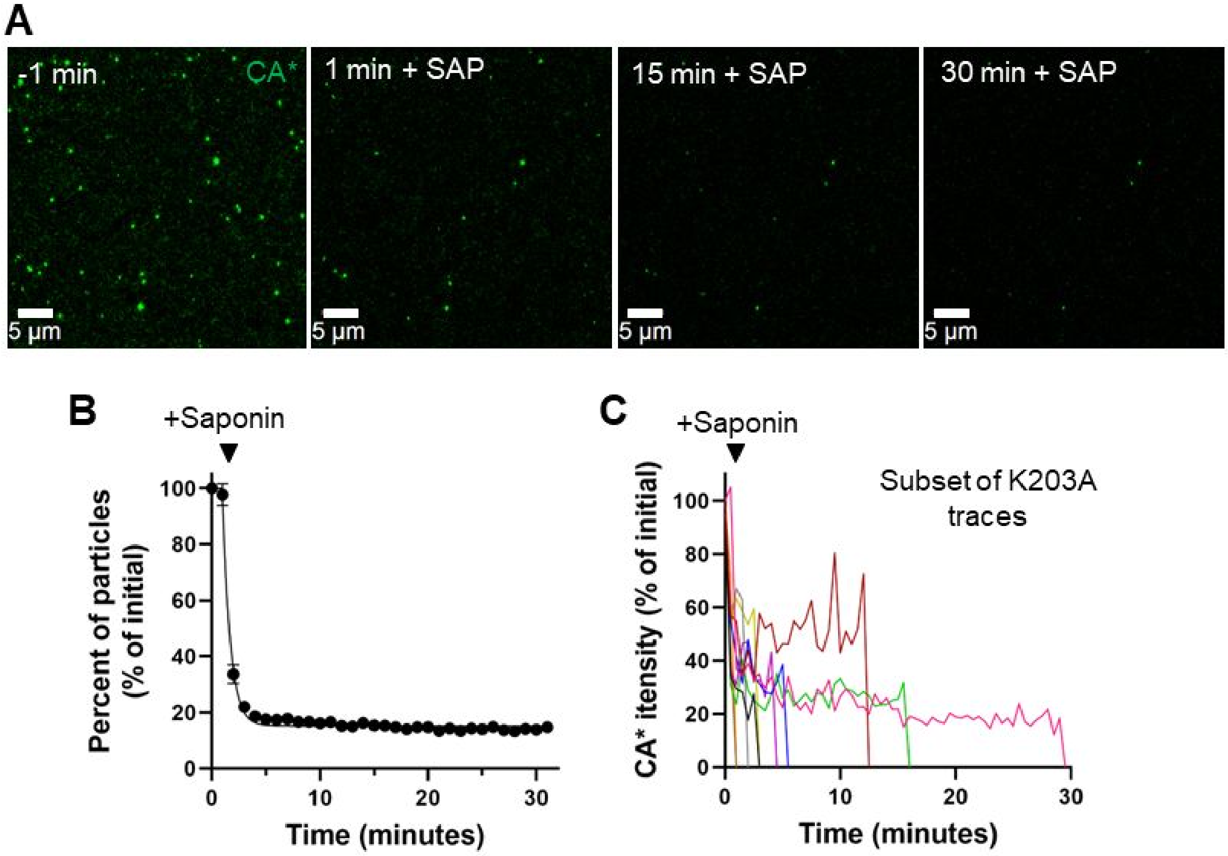

Fig. S3. K203A CA*/CA^{K203A} pseudoviruses are unstable *in vitro*. A) Representative micrographs of K203A CA* core uncoating *in vitro* using stringent imaging conditions (0.09 x 0.09 µm pixel size). Saponin (100 µg/mL) was added to coverslip adhered K203A CA* pseudoviruses, and virus membrane permeabilization and loss of CA* monitored by single particle tracking. Particles retaining CA* fluorescence at 1 min after saponin addition (1 min +SAP) represent K203A cores that did not uncoat immediately upon lysis. **B)** *In vitro* K203A CA* pseudovirus uncoating kinetics imaged as in panel D. Mean and SD from 2 independent experiments are plotted. Incomplete loss of CA* signal from a small fraction of labeled pseudoviruses by the end of experiment (30 min) is responsible for the non-zero plateau in the uncoating kinetics. **C)** A small subset (~15%) of single K203A CA* particle intensity traces from the *in vitro* uncoating experiments in Fig. 1H, demonstrating a delayed loss of K203A CA* signal after saponin lysis.

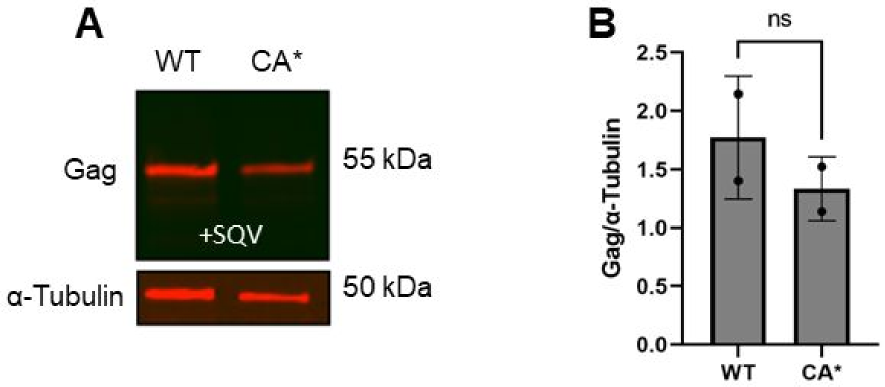

Fig. S5. Estimation of CA* and WT CA stoichiometry upon virus production. A) Fluorescent western blot showing SQV (200 nM) treated CA* and WT Gag from HEK293T/17 cells. CA* and WT Gag was detected using the human anti-HIV serum antibody and visualized with goat anti-human 800CW secondary antibody. GAPDH loading control is below the Gag western blot. GAPDH was detected using Rabbit anti-α Tubulin antibodies visualized with Goat anti-rabbit 800CW secondary antibody. **B)** Densitometry analysis of Gag/tubulin ratio is shown. Two-independent experiments with mean and S.D. plotted.

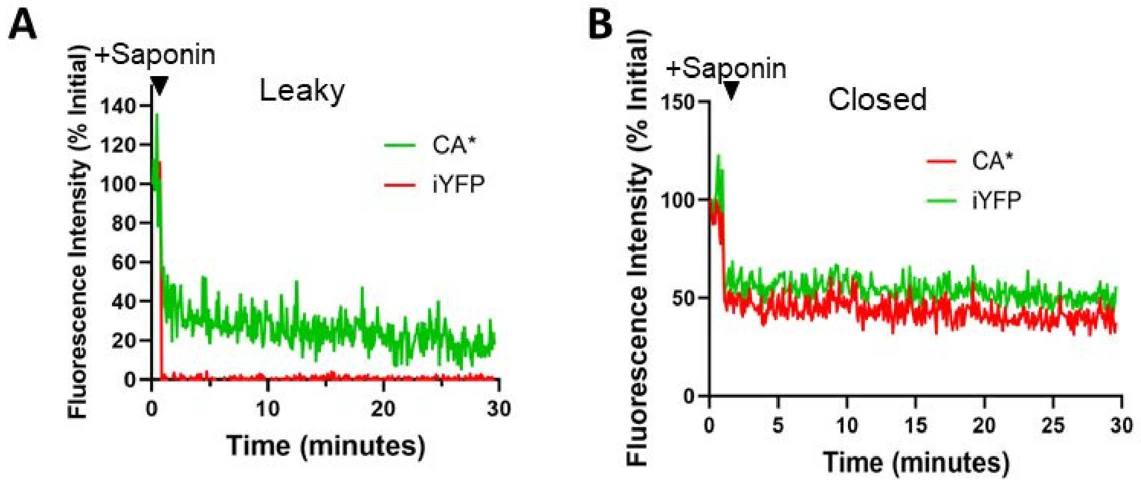

Fig. S6. Single particle intensity traces for leaky and closed CA*:Gag-iYFP cores *in vitro*. **A)** single particle intensity trace for coverglass-immobilized CA*:Gag-iYFP pseudovirus displaying the leaky iYFP loss phenotype. **B)** Single particle intensity trace for CA*:Gag-iYFP pseudovirus displaying the closed phenotype, never losing iYFP single from mature core.

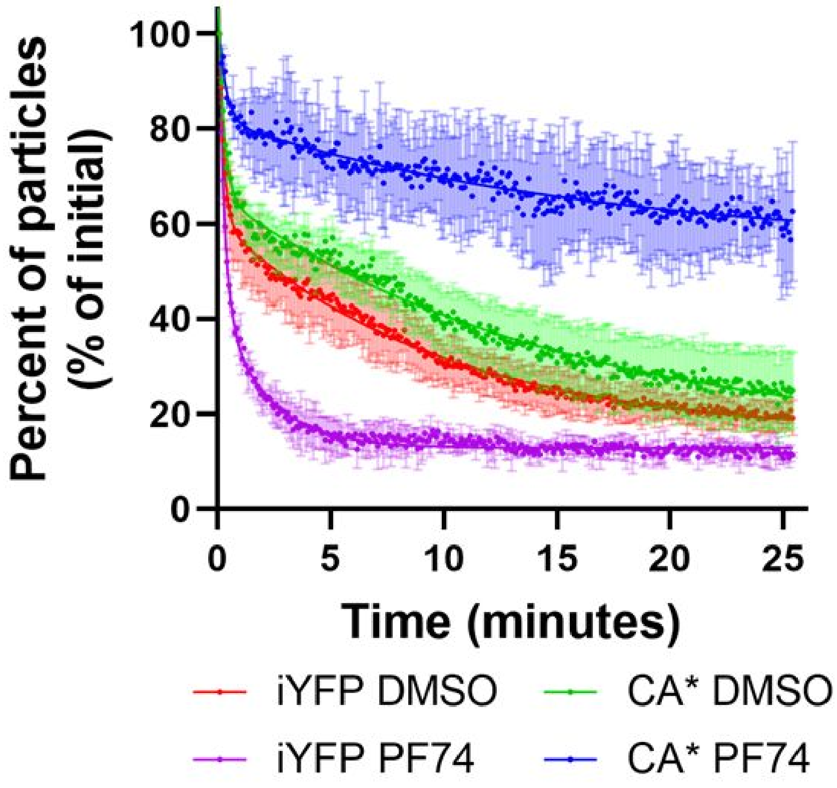

Fig. S7. Release of core-trapped iYFP from in vitro CA*:Gag-iYFP cores. Kinetics of uncoating (reduction in number of particles per image field) for CA*:Gag-iYFP cores *in vitro,* following saponin treatment (100 µg/mL, t=0) in the presence of 10 µM PF74 or DMSO. Particle counts were normalized to the initial quantity (Frame preceding saponin lysis). Mean and SD are plotted from 2 independent experiments.

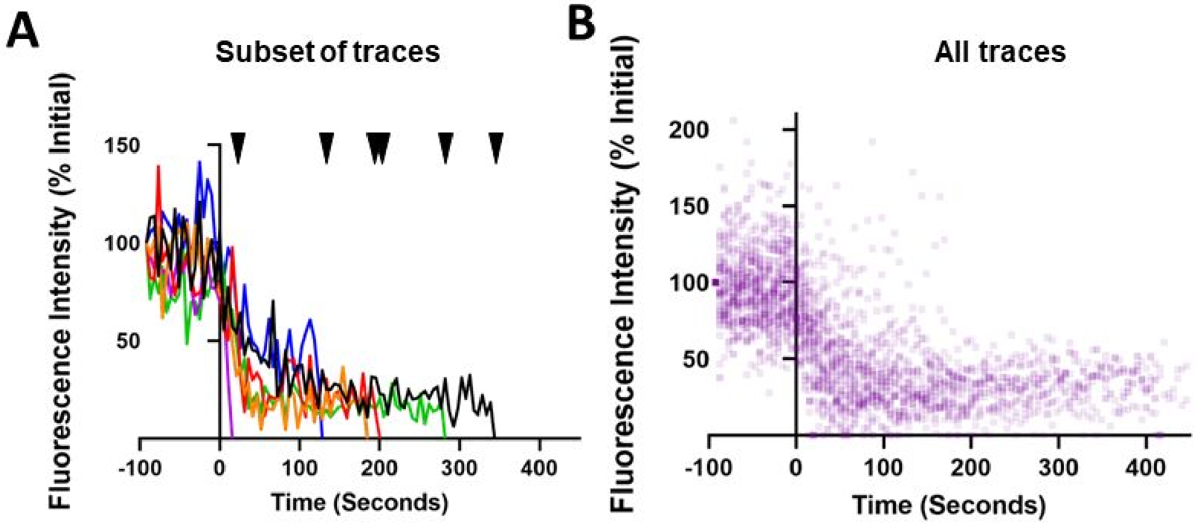

Fig. S8. Single particle intensity traces of all *in vitro* integrity loss and uncoating events from Fig. 4J. **A)** Single particle intensity traces from the ensemble plot in Fig, 4F. A small subset of traces was selected to demonstrate gradual CA* loss and residual CA* intensity after defect formation, all having an individual color. **B)** All traces were selected, and each time point is represented with a transparent point to demonstrate areas of overlap. Most particles undergo near-immediate loss of CA* fluorescence after integrity loss.

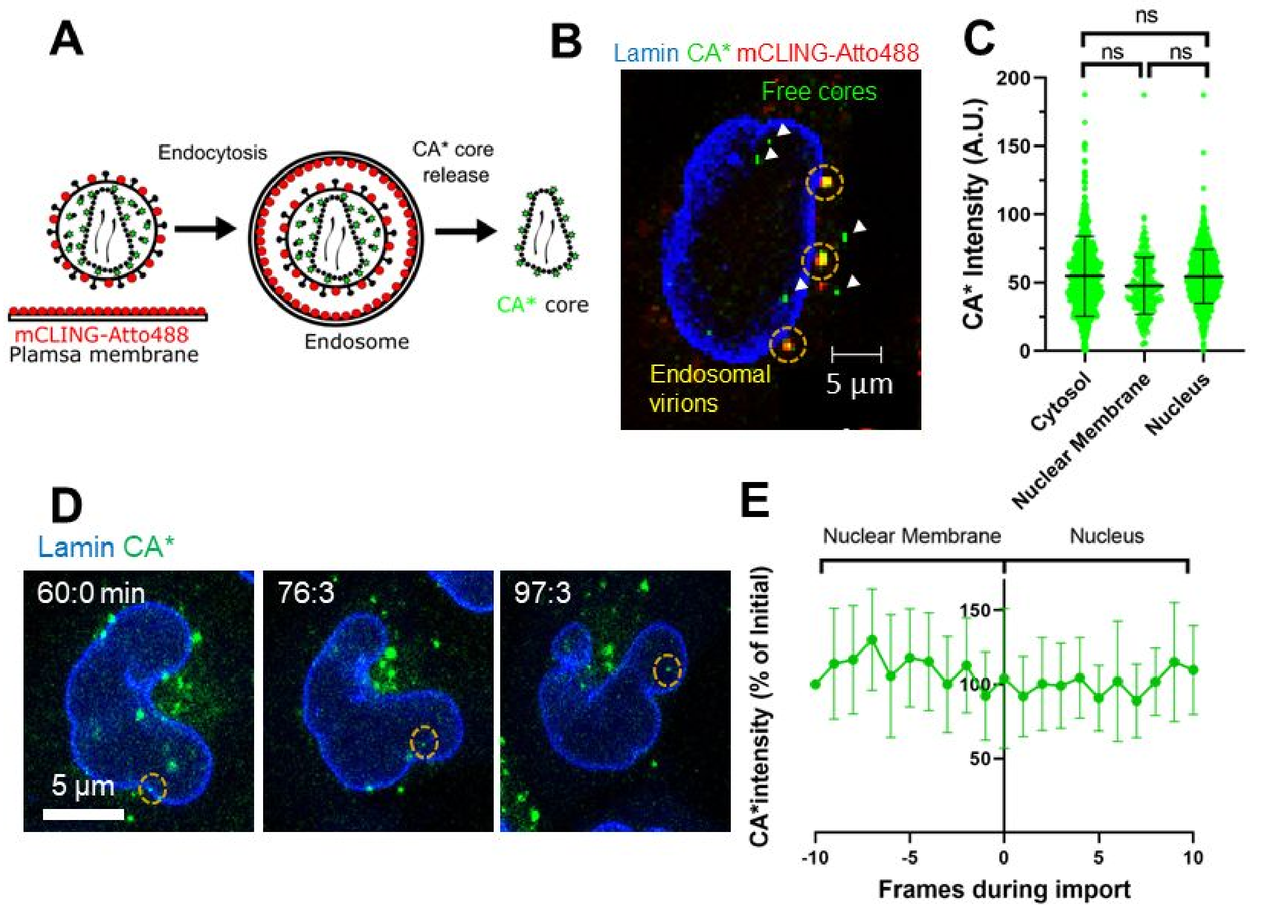

Fig S9. HIV-1 capsid protein is not lost upon nuclear entry into GHOST-SNAP-lamin cells. Cells were infected with CA* labeled particles pseudotyped by VSV-G that drives efficient HIV-1 pseudovirus entry through endocytic route.[85-87] Four hours post-infection (hpi), cells were fixed, and the fluorescence intensity distributions of single CA* cores in the cytosol, at the nuclear membrane, and in the nucleus were compared. **A)** Schematic illustration for mCLING-Atto488 labeling of GHOST-SNAP-lamin cells and CA* viral membranes to visualize endosome localized CA* pseudoviruses. **B)** Representative image of infected GHOST-SNAP-lamin cells having endosome localized CA* pseudoviruses (circled in yellow, colocalized with mCLING-Atto488 (red)) and several free CA* cores in the cytosol and nucleus (white arrows). SNAP-Lamin B1 was labeled with SNAP-TMR-Star. Image was taken at 4 hpi. **C)** Fluorescence intensity distributions of cytosolic, nuclear membrane and nucleus localized CA* cores at 4 hpi (Cytosolic: $n = 881$, Nuclear membrane: $n = 299$, Nucleus: $n = 622$) (Cytoplasm:Nucleus: $p = 0.995$, Nuclear membrane:Nucleus: $p = 0.777$, Cytoplasm:Nuclear membrane: $p = 0.927$, Kolmogorov-Smirnov test with optimal binning). **D)** Representative time-lapse micrograph of an individual CA* core entering the nucleus of a GHOST(3) SNAP-Lamin cell at 76.3 minutes post infection, SNAP-Lamin B1 labeled with SNAP-OregonGreen particle of interest is marked by yellow circle. Large perinuclear foci are likely unfused/immature CA* pseudoviruses that were internalized into the cell. Temporal resolution ranges from ~1 min per frame. **E)** Ensemble normalized fluorescence intensity profiles of 8 nuclear entry events aligned at the time of nuclear entry.

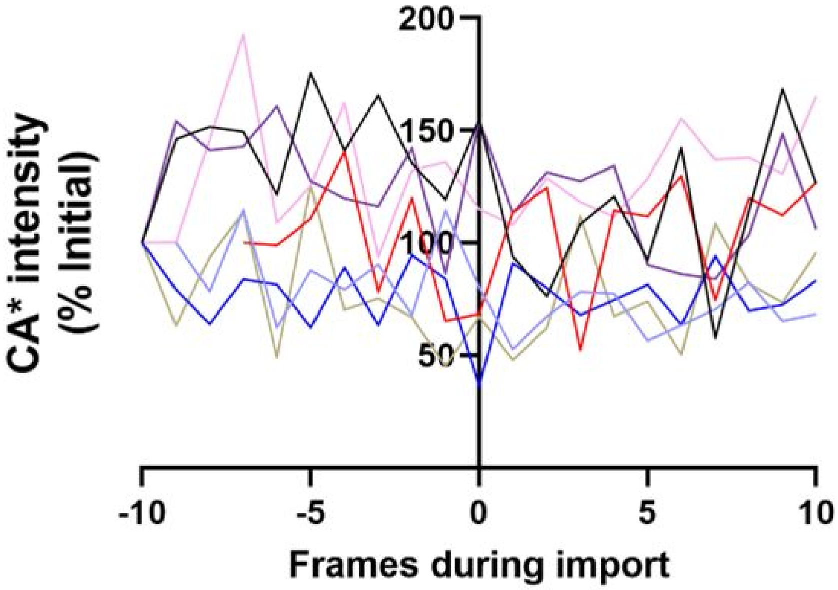

Fig. S10. Individual traces used in nuclear import CA* ensemble intensity trace from Fig. 2E. All eight single particle intensity traces used in Fig. 2E for ensemble intensity visualization of retained CA* during nuclear import. T = 0 represents nuclear import (IE intranuclear localization) of CA* cores.

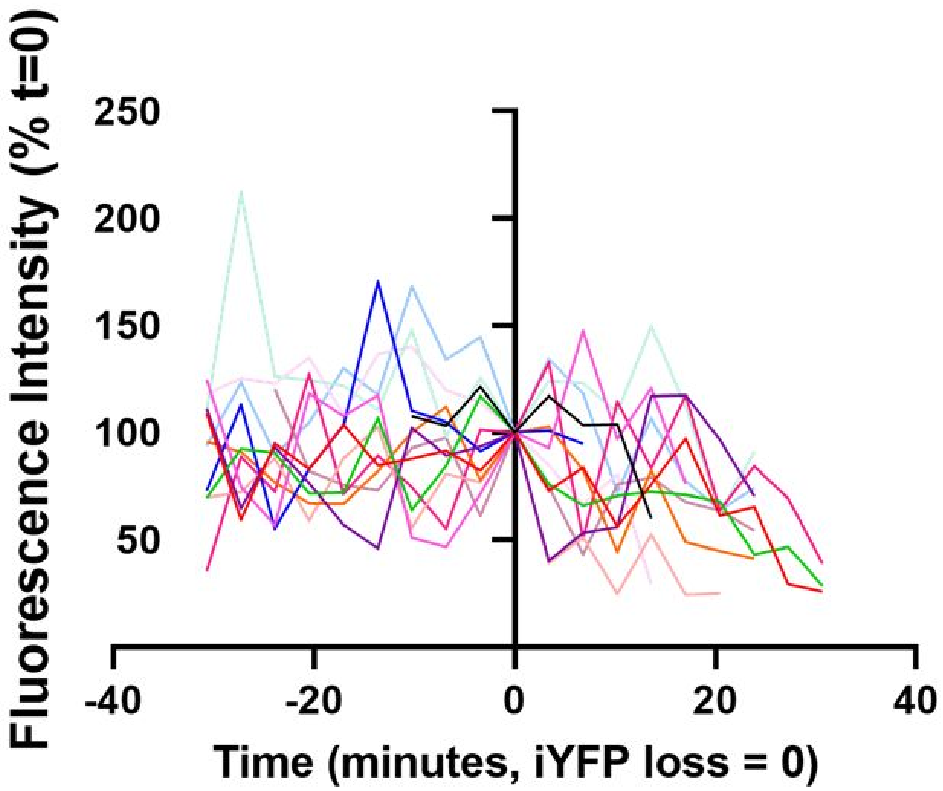

Fig. S11. Individual traces used in CA* uncoating ensemble intensity trace from Fig. 5I. All 18 single particle intensity traces used in Fig. 2E for ensemble intensity visualization of CA* intensity during iYFP loss and uncoating. T = 0 loss of capsid integrity (iYFP loss) from CA* cores.

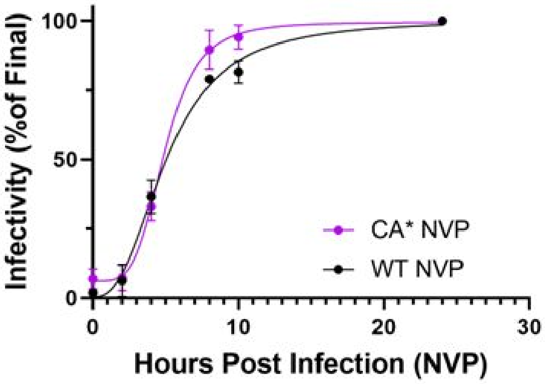

Fig. S12. NVP time-of-addition assay to assess reverse transcription kinetics. Representative reverse transcription kinetics of CA^{WT} and CA* pseudoviruses treated with 10 μM NVP at 0, 2, 4, 8, 10, 24 hpi. The T_{50} for WT and CA* pseudoviruses are 5.1 and 4.9 hpi, respectively. Mean and S.D. plotted between 2 independent virus preparations.

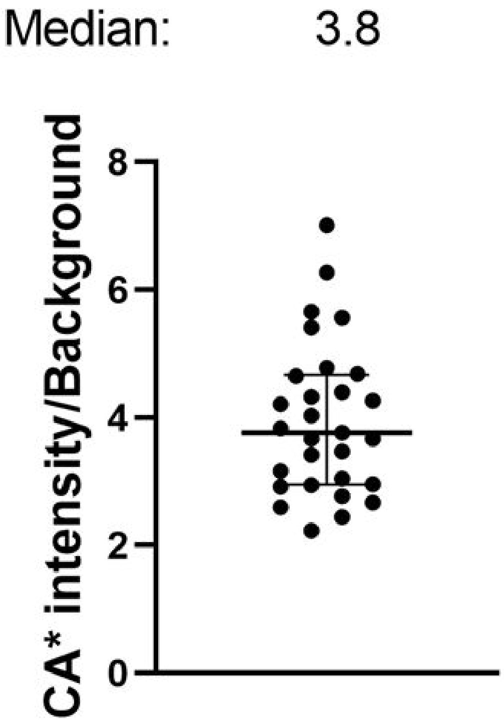

Fig. S13. Live cell imaging signal-to-background ratios of CA*:Gag-iYFP cores. Signal-to-noise ratios of 28 CA*:Gag-iYFP cores in the nucleus of GHOST-Lamin cells. Median and interquartile range are used for determination of median. Median value of signal-to-background for all 28 cores is listed above the graph.

Supplementary Movie Legends

Movie S1. Cytosolic uncoating progresses through two-steps. Representative movie of a single CA*:Gag-iYFP labeled core within the cytosol that loses of integrity (releases iYFP, red) and uncoats (loses CA*, green) within the cytosol of a GHOST-SNAP-Lamin cell. SNAP-Lamin B1 is labeled with SNAP-TMR-STAR. Arrows point to CA*:Gag-iYFP core of interest with annotations signifying the integrity loss and uncoating events.

Movie S2. Nuclear periphery-localized uncoating progresses through two-steps. Representative movie of a single CA*:Gag-iYFP labeled core at the nuclear periphery that loses of integrity (releases iYFP, red) and uncoats (loses CA*, green) at the nuclear pore of a GHOST-SNAP-Lamin cell. SNAP-Lamin B1 is labeled with SNAP-TMR-STAR. Arrows point to CA*:Gag-iYFP core of interest with annotations signifying the integrity loss and uncoating events.

Movie S3. Live cell imaging of CA* nuclear import. Representative movie of a single CA* core entering the nucleus of a GHOST-SNAP-Lamin cell at early times post infection without losing CA* fluorescence. SNAP-Lamin B1 is labeled with SNAP-OregonGreen. Red arrows point to cores of interest with annotations signifying the nuclear import event.

Movie S4. Multi-step nuclear HIV-1 uncoating in a GHOST-SNAP-Lamin cell. Representative movie of a single CA*:Gag-iYFP labeled core that loses of integrity (releases iYFP, red) and uncoats (loses CA*, green) within the nucleus of a GHOST-SNAP-Lamin cell. SNAP-Lamin B1 is labeled with SNAP-TMR-STAR. Arrows point to CA*:Gag-iYFP core of interest with annotations signifying the integrity loss and uncoating events.

Movie S5. PF74 leads to release of core-trapped fluid phase marker and results in capsid degradation. Representative movie showing PF74-induced loss of integrity (iYFP loss, red) of a single CA*:Gag-iYFP core followed by degradation of the capsid lattice (loss of CA* signal, green). SNAP-Lamin B1 is labeled with SNAP-TMR-STAR. Arrows point to the CA*:Gag-iYFP core of interest with annotations signifying the integrity loss and CA* degradation events. 10 μM PF74 added to cells.

Movie S6. Multi-step uncoating in the nucleus of THP-1 macrophage-like cells. Representative movie of a single CA*:Gag-iYFP core undergoing loss of integrity (release of iYFP, red) followed by terminal uncoating (loss of CA*, green) within the nucleus in a differentiated THP-1 macrophage cell. Nucleus is stained with Hoechst 33342 (blue). Arrows point to CA*:Gag-iYFP core of interest with annotations signifying the integrity loss and uncoating events.

Movie S7. Nuclear uncoating correlates with productive infection. Representative movie for terminal nuclear uncoating of a single CA* core (green) in the nucleus of a GHOST-SNAP-Lamin cell (MOI 0.1). SNAP-Lamin B1 is labeled with SNAP-TMR-STAR. Uncoating and GFP reporter expression (red) are annotated, and red arrow points to core of interest.
